# Supplementary material for: Nascent peptide-induced translation discontinuation in eukaryotes impacts biased amino acid usage in proteomes
Source: Nat Commun. 2022 Dec 2;13:7451. doi: 10.1038/s41467-022-35156-x (PMC9718836; doi:10.1038/s41467-022-35156-x)
Supplement: Supplementary file 1 — Supplementary Information [file 41467_2022_35156_MOESM1_ESM.pdf]

Supplementary Information

Nascent peptide-induced translation discontinuation in eukaryotes impacts biased amino acid usage in proteomes

Yosuke Ito, Yuhei Chadani, Tatsuya Niwa, Ayako Yamakawa, Kodai Machida, Hiroaki Imataka, and Hideki Taguchi

Supplementary Figure 1: Detailed analysis of translation discontinuation caused by negatively charged amino acids.

Supplementary Figure 2: Supporting data for D/E-runs-dependent translation discontinuation in eukaryotes evaluated by the HsPURE system.

Supplementary Figure 3: Additional experiments on the translation discontinuation caused by endogenous D/E-enriched sequences.

Supplementary Figure 4: Additional experiments on the function of Pth2p.

Supplementary Figure 5: Identification of the abortive peptidyl-tRNAs accumulated in the *pth2*Δ strain by LC-MS/MS analysis.

Supplementary Figure 6: Data analysis of yeast proteome sequences.

Supplementary Figure 7: Distribution of the D/E-enriched sequences among all kingdoms of life.

Supplementary Figure 8: Distribution of the 10 or more consecutive amino acid sequences among all kingdoms of life.

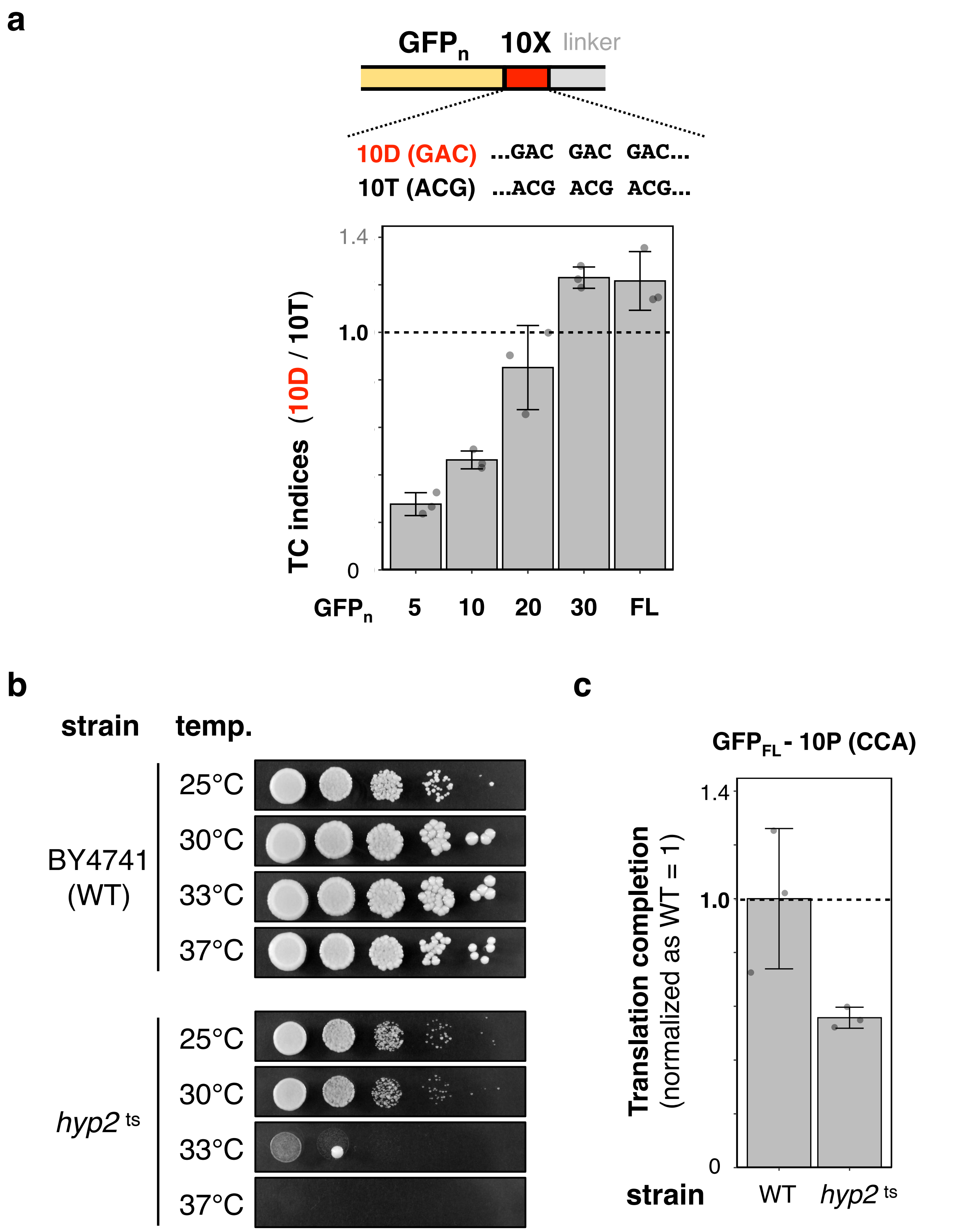

Supplementary Figure 1

Detailed analysis of translation discontinuation caused by negatively charged amino acids.

(**a**) Translation attenuation depends on the negatively charged amino acid sequence but not the nucleotide sequence in the vicinity of the N-terminal region *in vivo*. The TC indices were

calculated by comparing the relative Fluc activity between 10D (GAC) and 10T (ACG, -1 frameshift), as shown in **Fig. 1b**. Error bars indicate standard deviations from three biological replicates.

(**b and c**) Confirmation of *hyp2*^{ts} strain, TSA737. Wild-type (WT) and *hyp2*^{ts} strains were spotted on the YPD plate at indicating temperature for 2 days. The *hyp2*^{ts} strain shows a semi-lethal phenotype at 33°C (**b**) and the inefficiency of a poly-proline (10P) translation at 33°C (**c**). Error bars indicate standard deviations from three biological replicates.

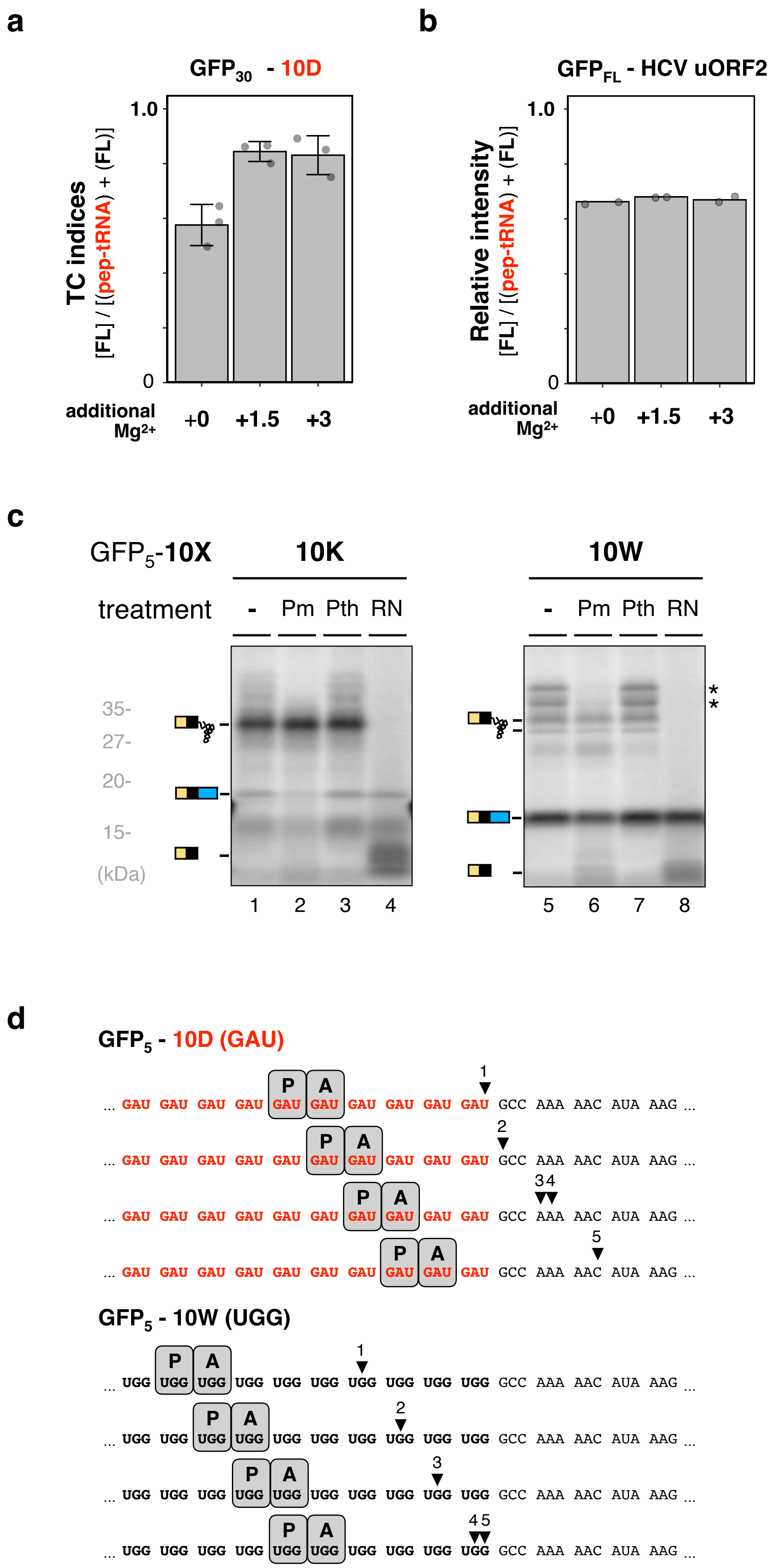
a
GFP30 - 10D
TC indices
[FL] / [(pep-tRNA) + (FL)]
1.0
0
additional Mg2+
+0
+1.5
+3
b
GFP_FL - HCV uORF2
Relative intensity
[FL] / [(pep-tRNA) + (FL)]
1.0
0
additional Mg2+
+0
+1.5
+3
c
GFP5-10X
10K
10W
treatment
-
Pm
Pth
RN
35-
27-
20-
15-
(kDa)
1
2
3
4
5
6
7
8
d
GFP5 - 10D (GAU)
GFP5 - 10W (UGG)
P
A
GCC AAA AAC AUA AAG

Supplementary Figure 2

Supporting data for D/E-runs-dependent translation discontinuation in eukaryotes evaluated by the HsPURE system.

(**a**) Effect of Mg^{2+} on the 10D sequence-dependent translation attenuation. The GFP_{30}-10D sequences were translated by the HsPURE system supplemented with additional Mg^{2+} as indicated, and individual TC indices were calculated. Error bars indicate standard deviations from three technical replicates.

(**b**) Effect of Mg^{2+} on the hCMV uORF-induced translational stalling. The relative intensity of the peptidyl-tRNAs calculated from two independent experiments was shown.

(**c**) The GFP_5-10K-Fluc (lanes 1-4) or GFP_5-10W-Fluc (lanes 5-8) template mRNA was translated using the HsPURE system including ^{35}S-methionine. The translated products were treated with puromycin (Pm) or peptidyl-tRNA hydrolase (yeast Pth2p: *Pth*) as indicated and separated by neutral pH SDS-PAGE with optional RNase A (*RN*) treatment. Radioactive bands were developed by using an imaging plate. Peptidyl-tRNAs, tRNA-cleaved polypeptides and full-length product are indicated by schematic labels. Asterisks indicate the peptidyl-tRNA which is accommodated within the ribosome stalled at the stop codon or 3' end of mRNA. Representatives of two technical replicates are shown.

(**d**) Schematic illustrations of the major sites of ribosome stalling on the GFP_5-10D and GFP_5-10W mRNA evaluated from the toeprint analysis in **Fig. 2d**. The points of reverse transcription interference are indicated by arrows, and the ribosomal occupancies are shown by the A-site and P-site codons.

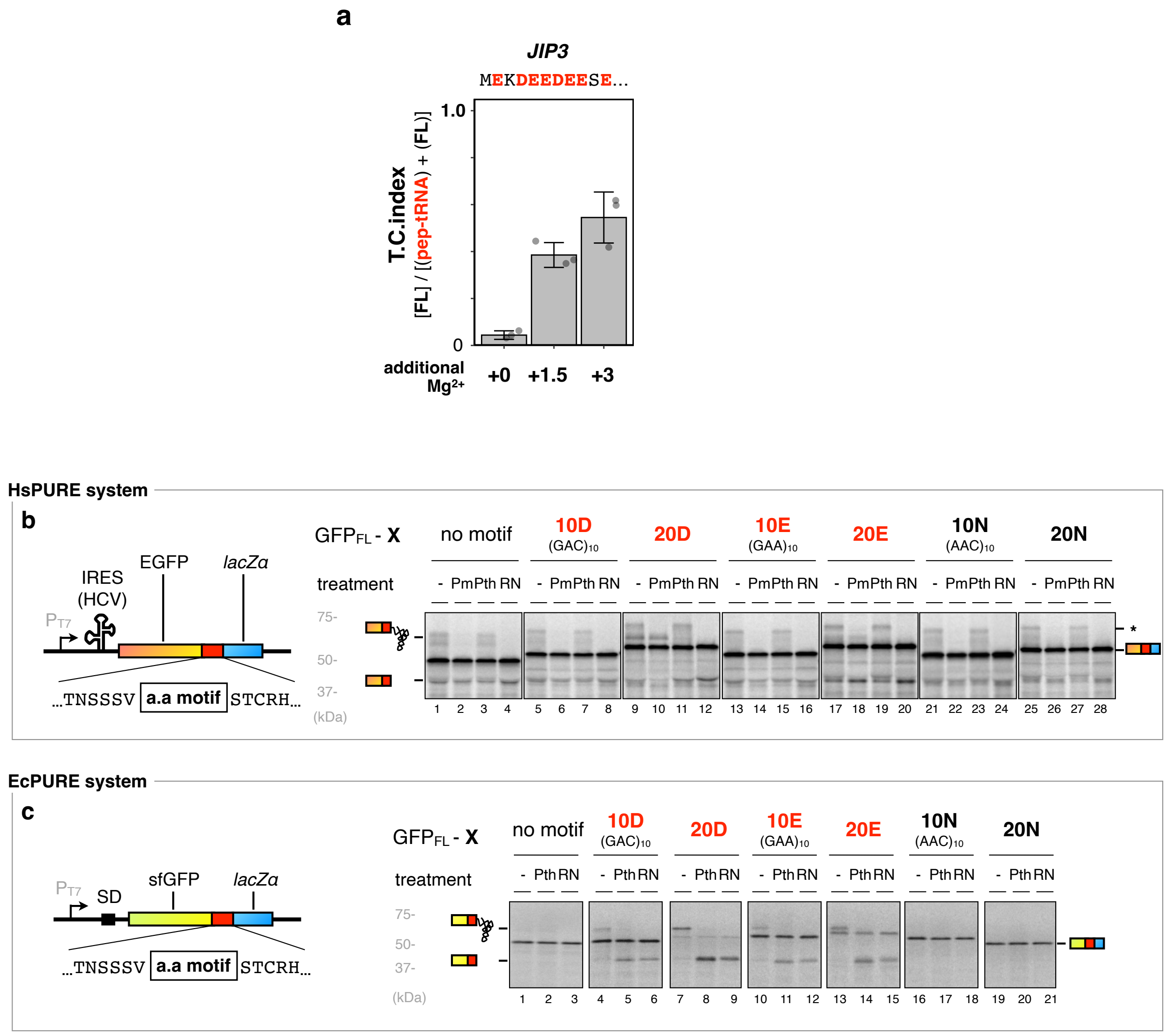

Supplementary Figure 3

Additional experiments on the translation discontinuation caused by endogenous D/E-enriched sequences.

(**a**) An endogenous D/E-rich sequence attenuates translation elongation in an Mg^{2+}-dependent manner. The fusion of the N-terminal 10 a.a. of *JIP3* and a truncated Fluc (**Fig. 3d**) was translated by the HsPURE system supplemented with additional Mg^{2+} as indicated, and individual TC indices were calculated, as shown in **Fig. 1f**. Error bars indicate standard deviations from three technical replicates.

(**b**) The EGFP-10X mRNAs were translated using the HsPURE system including ^{35}S-methionine as shown in **Fig. 1e**. The products were treated with a peptidyl-tRNA hydrolase (yeast Pth2p: *Pth*) or puromycin (Pm) as indicated and separated by neutral pH SDS-PAGE with optional RNase A (*RN*) treatment. Full-length products, peptidyl-tRNAs, and the tRNA-

cleaved polypeptides are indicated by schematic labels. Representatives of two technical replicates are shown.

(**c**) The sfGFP-10X mRNAs were translated using the *E. coli* factor-based reconstituted cell-free translation system (PURE*frex* v1.0, EcPURE) including ^{35}S-methionine. The products were treated with a peptidyl-tRNA hydrolase (*E. coli*: *Pth*) as indicated and separated by neutral pH SDS-PAGE with optional RNase A (*RN*) treatment. Representatives of two technical replicates are shown.

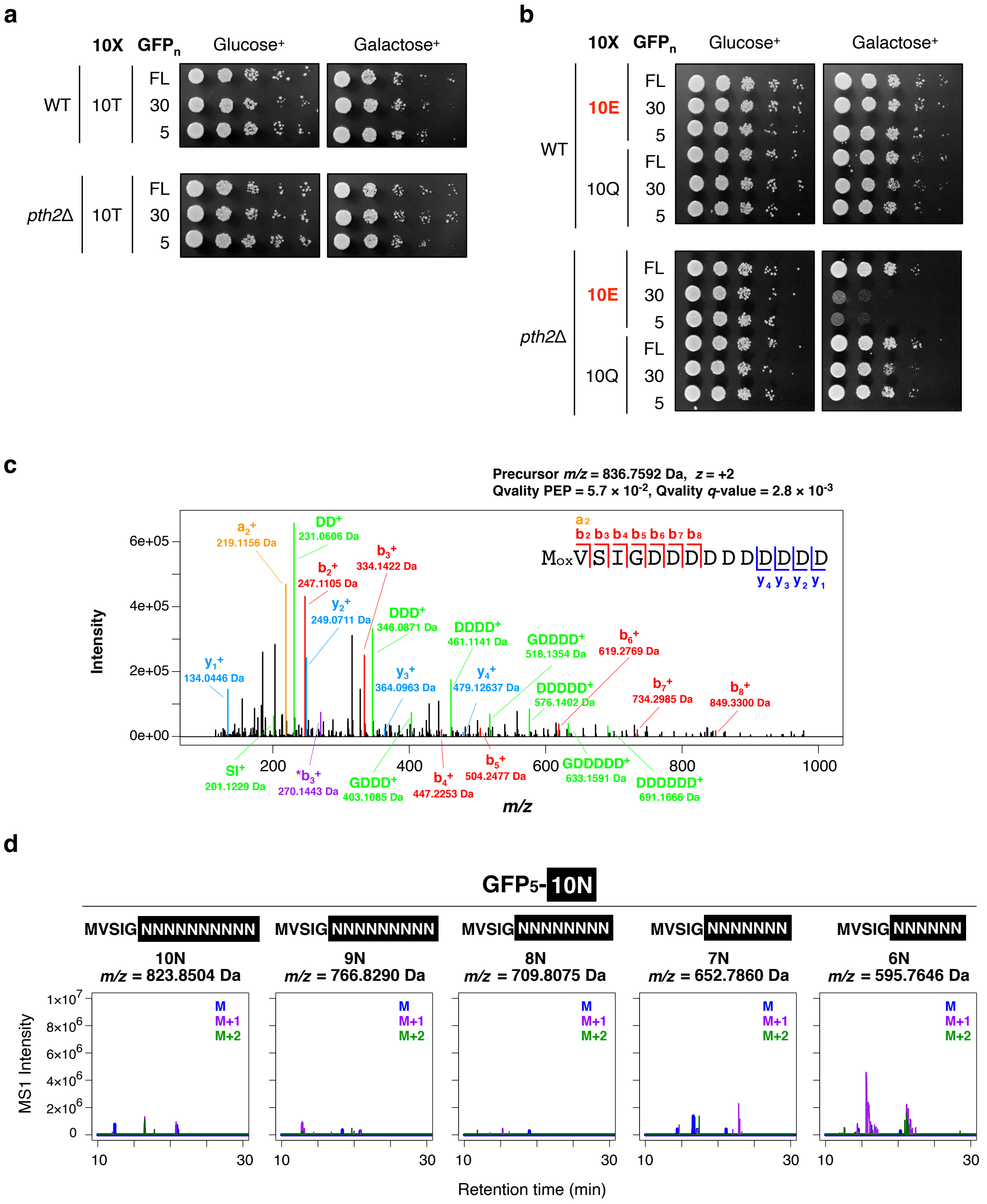

Supplementary Figure 4

Additional experiments on the function of Pth2p.

(**A**) Wild-type (WT) and *pth2*Δ strains harboring the plasmids for the expression of the GFPX-10T shown in **Supplementary Fig. 1a** were spotted on SD or SG plate lacking uracil at 30°C for 2-3 days.

(**b**) Wild-type (WT) and *pth2*Δ strains harboring inducible plasmids shown in **Fig. 1b** (10E or 10Q series) were spotted on SD or SG plate lacking uracil at 30°C for 2-3 days.

(**c**) The MS/MS spectrum of the M_{ox}VSIGDDDDDDDD peptide fragment expressed from GFP_5-10D. The peaks of the a-, b-, and y- fragment ions are shown in orange, red and blue, respectively. The major peaks derived from the internal fragments are shown in green. The above annotations are obtained by Proteome Discoverer 2.4. The peak of the b_3 ion with neutral losses of oxidized methionine (-CH_3SOH; -63.9983 Da) is also annotated manually (< 10 ppm from corresponding theoretical molecular masses), and shown in purple. Qvality PEP score and *q*-value are calculated by the Percolator algorithm on Proteome Discoverer 2.4.

(**d**) Extracted ion chromatograms of the peptide fragment expressed from the GFP_5-10N sequence in yeast *pth2*Δ cells. Each panel represents extracted ion chromatograms of MS1 intensity derived from a GFP_5-(X)N peptide derived from the peptidyl-tRNA. A chromatogram of monoisotopic ion is depicted in blue, and its +1 and +2 isotopes are depicted in purple and green, respectively. The specific *m/z* value of the monoisotopic ion is shown at the top.

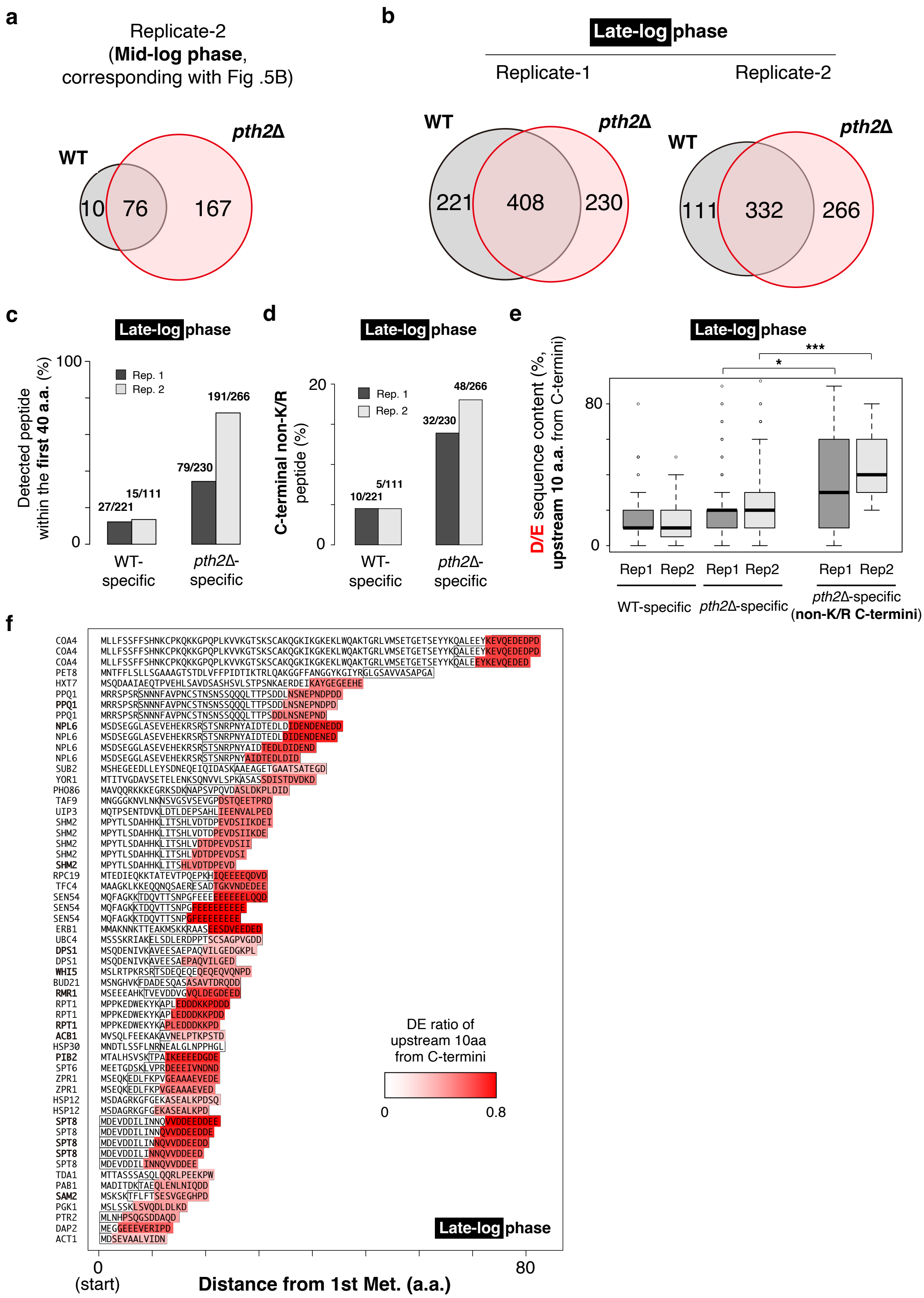
a
Replicate-2
(Mid-log phase,
corresponding with Fig .5B)
WT
pth2Δ
10
76
167
b
Late-log phase
Replicate-1
Replicate-2
WT
pth2Δ
221
408
230
111
332
266
c
Late-log phase
Detected peptide within the first 40 a.a. (%)
Rep. 1
Rep. 2
27/221
15/111
79/230
191/266
WT-specific
pth2Δ-specific
d
Late-log phase
C-terminal non-K/R peptide (%)
10/221
5/111
32/230
48/266
e
Late-log phase
D/E sequence content (%, upstream 10 a.a. from C-termini)
Rep1 Rep2
WT-specific
pth2Δ-specific
pth2Δ-specific (non-K/R C-termini)
f
DE ratio of upstream 10aa from C-termini
Late-log phase
0 (start)
80
Distance from 1st Met. (a.a.)

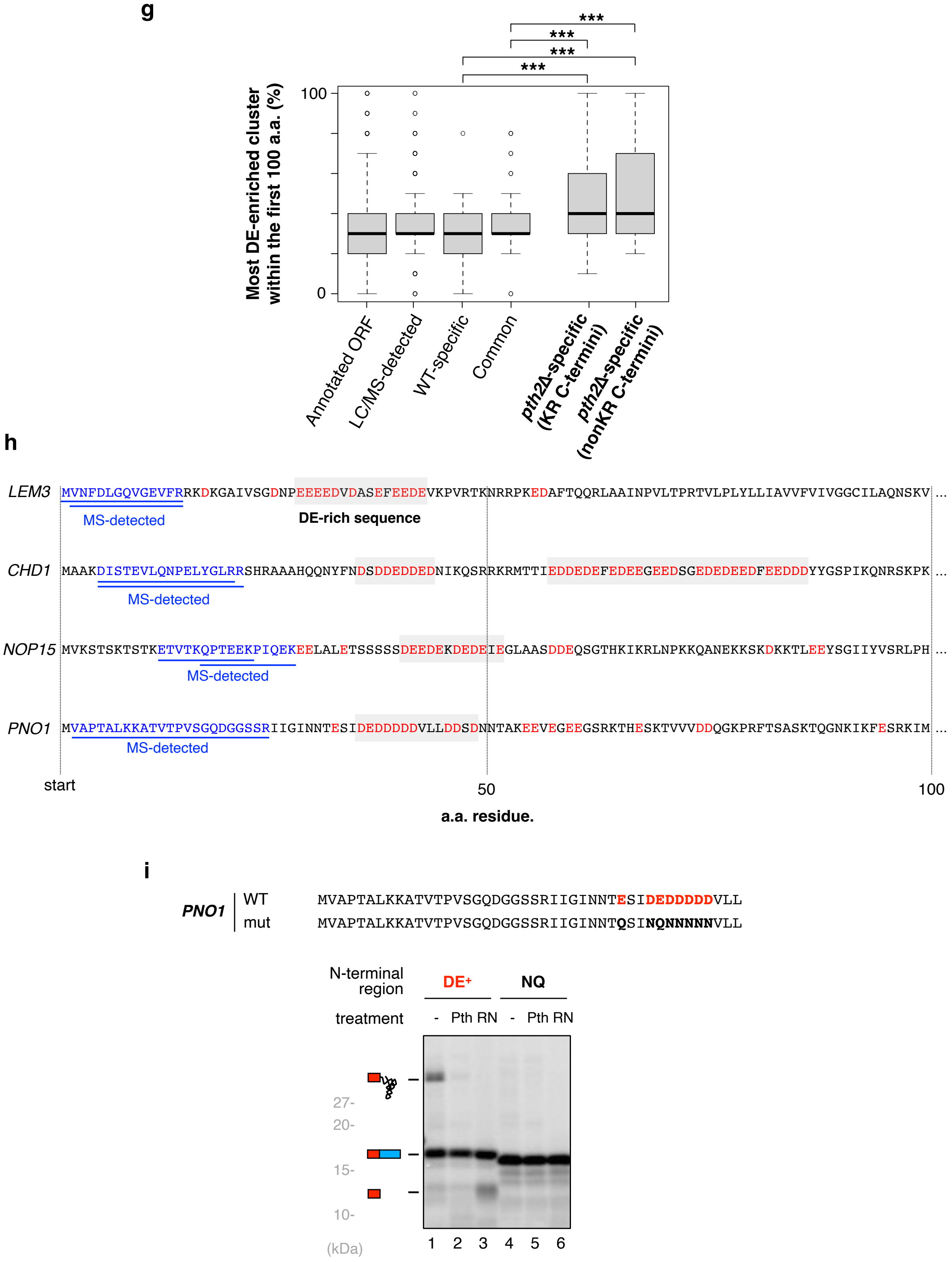

g
Most DE-enriched cluster within the first 100 a.a. (%)
100
0
***
***
***
***
Annotated ORF
LC/MS-detected
WT-specific
Common
pth2Δ-specific (KR C-termini)
pth2Δ-specific (nonKR C-termini)
h
LEM3 MVNFDLGQVGEVFRRKDKGAIVSGDNPEEEEDVDASEFEEDEVKPVRTKNRRPKEDAFTQQRLAAINPVLTPRTVLPLYLLIAVVFVIVGGCILAQNSKV ...
MS-detected
DE-rich sequence
CHD1 MAAKDISTEVLQNPELYGLRRSHRAAAHQQNYFNDSDDEDDEDNIKQSRRKRMTTIEDDEDEFEDEEGEEDSGEDEDEEDFEEDDDYYGSPIKQNRSKPK ...
MS-detected
NOP15 MVKSTSKTSTKETVTKQPTEEKPIQEKEELALETSSSSSDEEDEKDEDEIEGLAASDDEQSGTHKIKRLNPKKQANEKKSKDKKTLEEYSGIIYVSRLPH ...
MS-detected
PNO1 MVAPTALKKATVTPVSGQDGGSSRIIGINNTESIDEDDDDDVLLDDSDNNTAKEEVEGEEGSRKTHESKTVVVDDQGKPRFTSASKTQGNKIKFESRKIM ...
MS-detected
start
50
100
a.a. residue.
i
PNO1
WT MVAPTALKKATVTPVSGQDGGSSRIIGINNTESIDEDDDDDVLL
mut MVAPTALKKATVTPVSGQDGGSSRIIGINNTQSINQNNNNNVLL
N-terminal region
DE+
NQ
treatment
- Pth RN
- Pth RN
27-
20-
15-
10-
(kDa)
1 2 3 4 5 6

Supplementary Figure 5.

Identification of the abortive peptidyl-tRNAs accumulated in the *pth2*Δ strain by LC-MS/MS analysis.

(**a**) The numbers of peptides identified in each strain prepared from another biological replicate of mid-log culture and their overlap are represented in the Venn diagram.

(**b**) The numbers of peptides identified in each strain prepared from late-log culture and their overlap are represented in the Venn diagram. The results of the two biological replicates are shown.

(**c**) The ratio of WT-specific and *pth2*Δ-specific peptides whose C-terminal position was within 40 amino acids from the first methionine. The results of the two biological replicates from late-log cultured cells are shown. The numbers above the bar represent the number of all detected peptides and the peptides within the first 40 amino acids.

(**d**) The ratio of WT-specific and *pth2*Δ-specific peptides whose C-terminal amino acid was not Lys/Arg (C-terminal non-K/R). The results of the two biological replicates from late-log cultured cells are shown. The numbers above the bar represent the number of all detected peptides and the C-terminal non-K/R peptides.

(**e**) The distribution of the relative content of D/E residues in the upstream ten amino acids from the C-terminus of the detected peptides. The results of the two biological replicates from late-log cultured cells are shown. The box portions and the central bands are described according to the 25th percentile and the median, respectively. The whiskers indicate the maximum and minimum values excluding outliers defined by 1.5 times the length of the box. * $P < 0.01$, *** $P < 0.001$, by Wilcoxon's rank sum test (two-sided). The exact *P*-values are as follows; 0.001543 between replicate 1 of *pth2*Δ-specific and *pth2*Δ-specific and non-K/R C-termini and 2.335×10^{-12} between replicate 2 of *pth2*Δ-specific and *pth2*Δ-specific and non-K/R C-termini.

(**f**) Mapping of the identified C-terminal non-K/R peptides specific to the *pth2*Δ strain prepared from late-log cultured cells. Box represents the identified peptides by LC-MS/MS. The relative contents of D/E residues in the upstream ten amino acids from the C-terminus of the detected peptides are shown as a red gradient. A bold letter in the gene name indicates that the corresponding peptide was identified from both two biological replicates.

(**g**) The distribution of the maximum ratio of D/E in ten amino acid windows within N-terminal 100 amino acids. The box portions and the central bands are described according to the 25th percentile and the median, respectively. The whiskers indicate the maximum and minimum values excluding outliers defined by 1.5 times the length of the box (default settings of the "boxplot" function in R.app). The number of proteins in each group was as follows; *n*=6,713 for all annotated ORFs, *n*=1,336 for LC/MS-detected all, *n*=45 for WT-specific, *n*=96 for

common, *n*=121 for *pth2*Δ-specific and K/R C-termini, and n *n*=37 for *pth2*Δ-specific and non-K/R C-termini. *** $P < 0.001$, by Wilcoxon's rank sum test (two-sided). The exact *P*-values are as follows; 1.44×10^{-9} between WT-specific and *pth2*Δ-specific and K/R C-termini, 5.807×10^{-7} between WT-specific and *pth2*Δ-specific and non-K/R C-termini, 1.864×10^{-8} between common and *pth2*Δ-specific and K/R C-termini, and 1.543×10^{-5} between common and *pth2*Δ-specific and non-K/R C-termini.

(**h**) Examples of D/E-rich sequences downstream of detected *pth2*Δ-specific C-terminal K/R peptides. Blue letters and underlines indicate the detected peptide fragments by LC-MS/MS, and red letters represent D/E residues.

(**i**) IRD during the translation of endogenous *PNO1* depending on the D/E-rich sequences in the vicinity of the N-terminal region. The N-terminal region of *PNO1* including the D/E-rich sequence and its derivative (**upper**) were fused with Fluc and were translated using the HsPURE system (**bottom**), as described above. Representative of two technical replicates is shown.

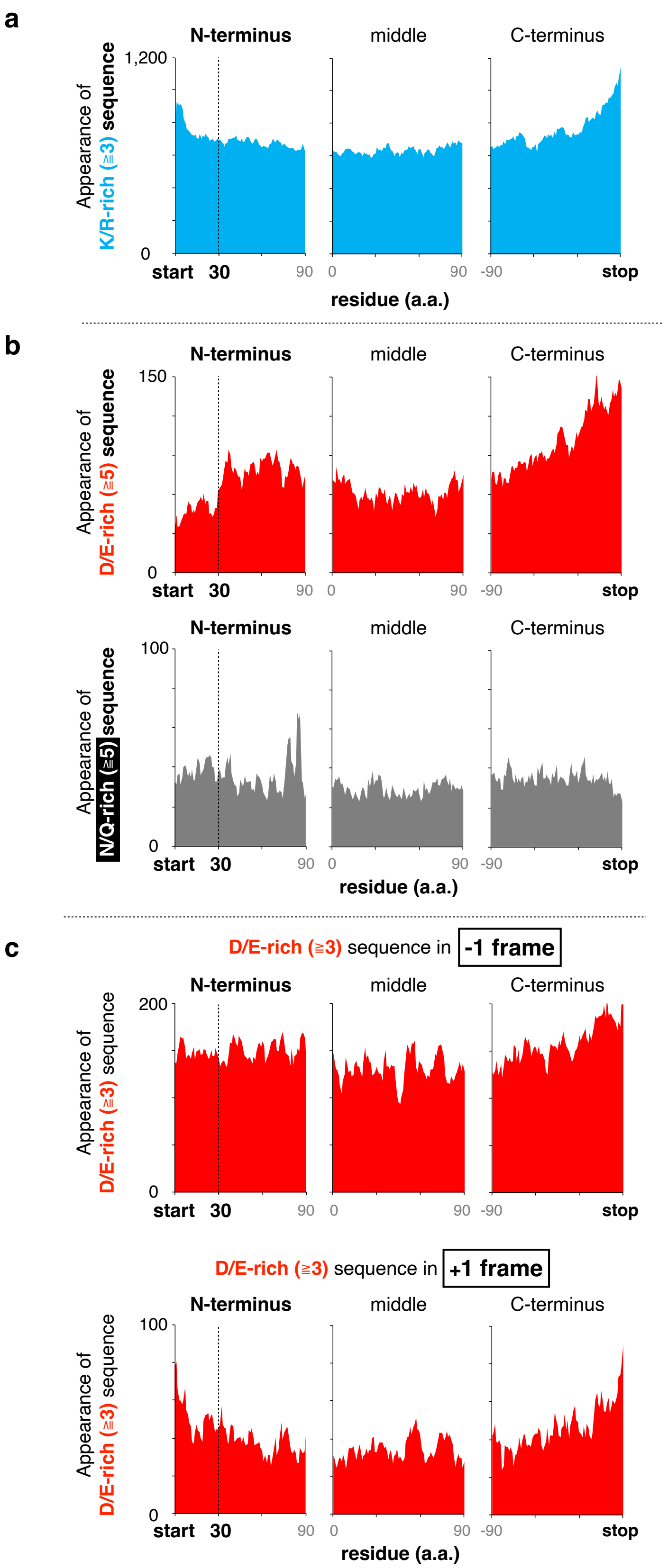

a
N-terminus
middle
C-terminus
Appearance of K/R-rich (≧3) sequence
1,200
0
start 30 90
0 90
-90 stop
residue (a.a.)
b
N-terminus
middle
C-terminus
Appearance of D/E-rich (≧5) sequence
150
0
start 30 90
0 90
-90 stop
N-terminus
middle
C-terminus
Appearance of N/Q-rich (≧5) sequence
100
0
start 30 90
0 90
-90 stop
residue (a.a.)
c
D/E-rich (≧3) sequence in -1 frame
N-terminus
middle
C-terminus
Appearance of D/E-rich (≧3) sequence
200
0
start 30 90
0 90
-90 stop
D/E-rich (≧3) sequence in +1 frame
N-terminus
middle
C-terminus
Appearance of D/E-rich (≧3) sequence
100
0
start 30 90
0 90
-90 stop
residue (a.a.)

Supplementary Figure 6

Data analysis of yeast proteome sequences.

(**a**) The composition of positively charged amino acids in the yeast proteome. The vertical axis represents the number of proteins harboring ≥3 K/R in a 10 residue-moving window. N-terminus, C-terminus, and middle region are defined as N-terminal 100 amino acids excluding the first methionine, C-terminal 100 amino acids, and middle 100 amino acids of the yeast 5,540 yeast proteins that have lengths greater than 130 amino acids.

(**b**) The number of proteins harboring ≥5 D/E (**upper**) or N/Q (**bottom**) in a 10 residue-moving window.

(**C**) Amino acids composition of the yeast proteome to confirm independency of a nucleic acid sequence bias. The vertical axis represents the number of proteins harboring ≥3 D/E in a -1 frameshift window (**upper**) or a +1 frameshift window (**bottom**).

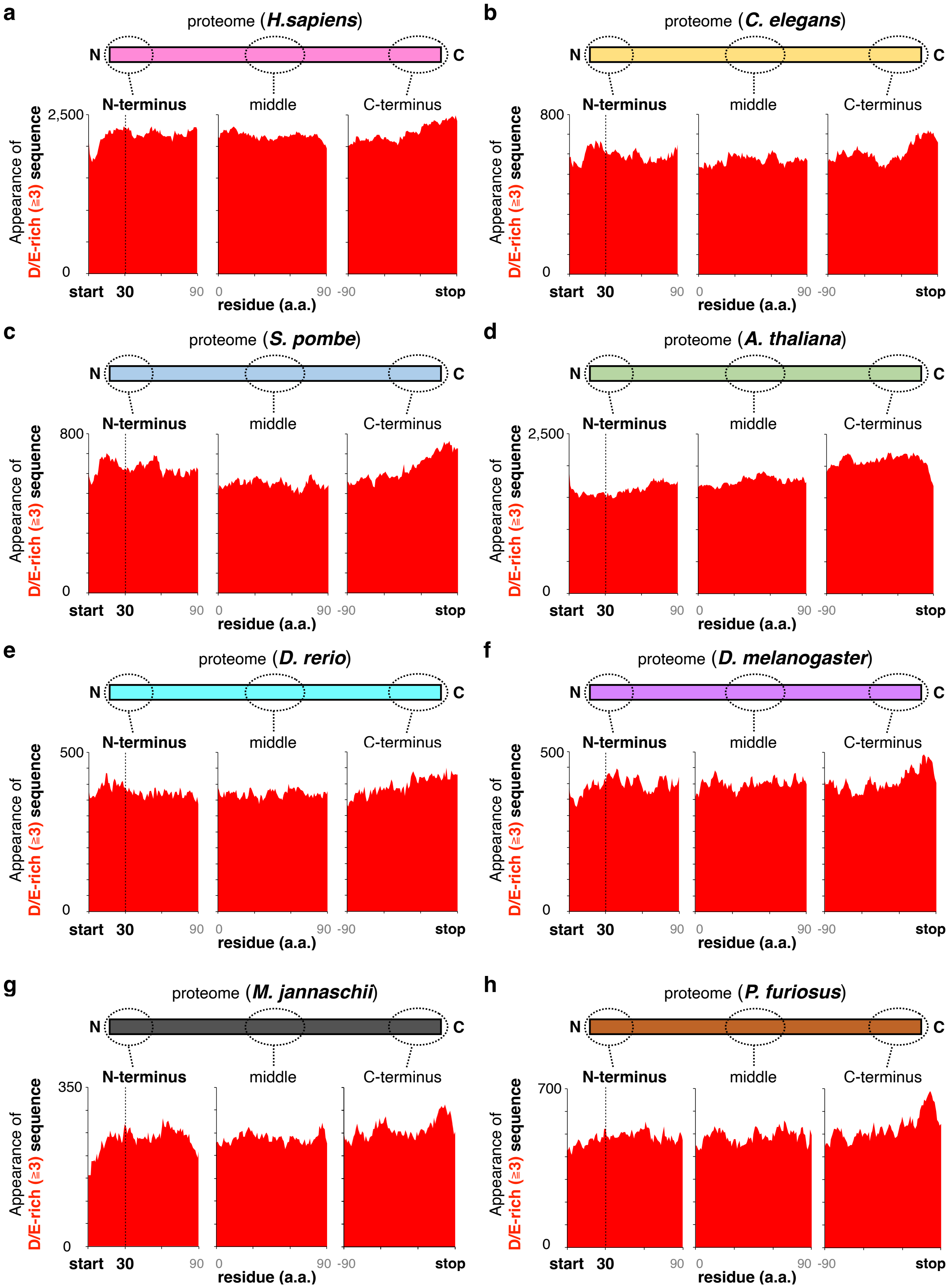

a
proteome (H.sapiens)
b
proteome (C. elegans)
c
proteome (S. pombe)
d
proteome (A. thaliana)
e
proteome (D. rerio)
f
proteome (D. melanogaster)
g
proteome (M. jannaschii)
h
proteome (P. furiosus)
N
C
N-terminus
middle
C-terminus
Appearance of
D/E-rich (≥3) sequence
start
30
90
0
-90
stop
residue (a.a.)
2,500
800
500
350
700

Supplementary Figure 7

Distribution of the D/E-enriched sequences among all kingdoms of life.

(**a-h**) Amino acids composition of the proteome in the various organisms. The number of gene having three or more D/E in a 10 residue-moving window. The vertical axis represents the number of protein harboring ≥3 target amino acids in a window. **a**. *Homo sapiens*; **b**. *Caenorhabditis elegans*; **c**. *Schizosaccharomyces pombe*; **d**. *Arabidopsis thaliana*; **e**. *Danio rerio*; **f**. *Drosophila melanogaster*; **g**. *Methanocaldococcus jannaschii* (archaea); **h**. *Pyrococcus furiosus* (archaea, only this proteome includes the dataset unreviewed by Swiss-Prot).

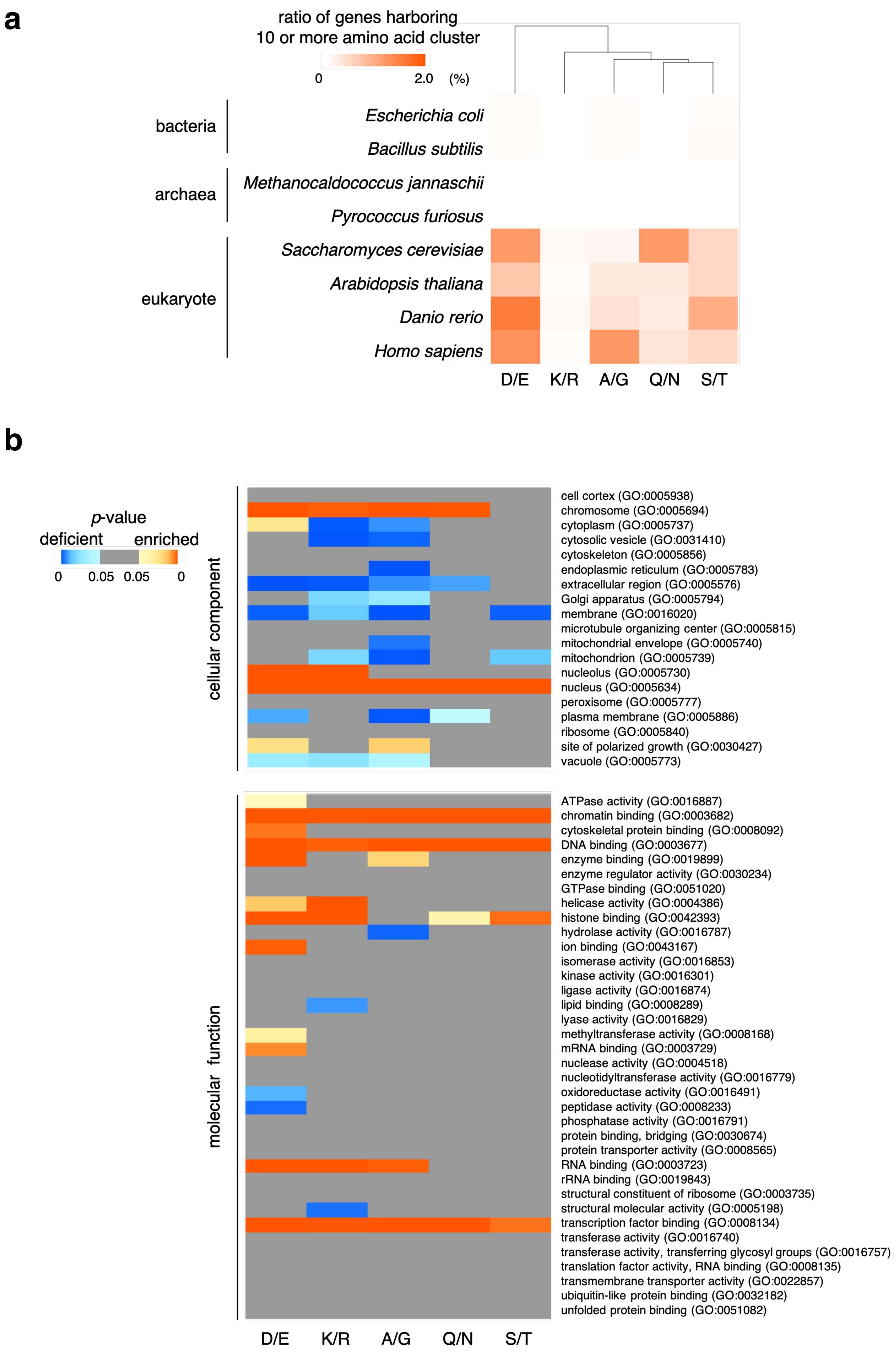
a
ratio of genes harboring
10 or more amino acid cluster
0
2.0
(%)
bacteria
archaea
eukaryote
Escherichia coli
Bacillus subtilis
Methanocaldococcus jannaschii
Pyrococcus furiosus
Saccharomyces cerevisiae
Arabidopsis thaliana
Danio rerio
Homo sapiens
D/E
K/R
A/G
Q/N
S/T
b
p-value
deficient
enriched
0
0.05
0.05
0
cellular component
cell cortex (GO:0005938)
chromosome (GO:0005694)
cytoplasm (GO:0005737)
cytosolic vesicle (GO:0031410)
cytoskeleton (GO:0005856)
endoplasmic reticulum (GO:0005783)
extracellular region (GO:0005576)
Golgi apparatus (GO:0005794)
membrane (GO:0016020)
microtubule organizing center (GO:0005815)
mitochondrial envelope (GO:0005740)
mitochondrion (GO:0005739)
nucleolus (GO:0005730)
nucleus (GO:0005634)
peroxisome (GO:0005777)
plasma membrane (GO:0005886)
ribosome (GO:0005840)
site of polarized growth (GO:0030427)
vacuole (GO:0005773)
molecular function
ATPase activity (GO:0016887)
chromatin binding (GO:0003682)
cytoskeletal protein binding (GO:0008092)
DNA binding (GO:0003677)
enzyme binding (GO:0019899)
enzyme regulator activity (GO:0030234)
GTPase binding (GO:0051020)
helicase activity (GO:0004386)
histone binding (GO:0042393)
hydrolase activity (GO:0016787)
ion binding (GO:0043167)
isomerase activity (GO:0016853)
kinase activity (GO:0016301)
ligase activity (GO:0016874)
lipid binding (GO:0008289)
lyase activity (GO:0016829)
methyltransferase activity (GO:0008168)
mRNA binding (GO:0003729)
nuclease activity (GO:0004518)
nucleotidyltransferase activity (GO:0016779)
oxidoreductase activity (GO:0016491)
peptidase activity (GO:0008233)
phosphatase activity (GO:0016791)
protein binding, bridging (GO:0030674)
protein transporter activity (GO:0008565)
RNA binding (GO:0003723)
rRNA binding (GO:0019843)
structural constituent of ribosome (GO:0003735)
structural molecular activity (GO:0005198)
transcription factor binding (GO:0008134)
transferase activity (GO:0016740)
transferase activity, transferring glycosyl groups (GO:0016757)
translation factor activity, RNA binding (GO:0008135)
transmembrane transporter activity (GO:0022857)
ubiquitin-like protein binding (GO:0032182)
unfolded protein binding (GO:0051082)
D/E
K/R
A/G
Q/N
S/T

Supplementary Figure 8

Distribution of the 10 or more consecutive amino acid sequences among all kingdoms of life.

(**a**) Frequency of the genes containing ten or more consecutive amino acid sequences in bacteria, archaea, and eukaryotes. Frequency was indicated by the intensity of the orange color.

(**b**) Gene ontology enrichment analysis for the human ORFs containing the ten or more consecutive amino acid sequences. The enriched or deficient terms are indicated by warm or cold colors, respectively.
