# Supplementary material for: Nascent peptide-induced translation discontinuation in eukaryotes impacts biased amino acid usage in proteomes
Source: Nat Commun. 2022 Dec 2;13:7451. doi: 10.1038/s41467-022-35156-x (PMC9718836; doi:10.1038/s41467-022-35156-x)
Supplement: Supplementary file 3 — Description of Additional Supplementary Files [file 41467_2022_35156_MOESM3_ESM.pdf]

Description of Additional Supplementary Files

File Name: Supplementary Data 1
Description: MS-identified peptides derived from peptidyl-tRNAs (Fig. 5 and Supplementary Fig. 5).

File Name: Supplementary Data 2
Description: Strains used in this study.

File Name: Supplementary Data 3
Description: Plasmids used in this study.

File Name: Supplementary Data 4
Description: Oligonucleotides used in this study.

File Name: Supplementary Data 5
Description: Settings of Proteome Discoverer 2.4
