# Supplementary material for: Nascent peptide-induced translation discontinuation in eukaryotes impacts biased amino acid usage in proteomes
Source: Nat Commun. 2022 Dec 2;13:7451. doi: 10.1038/s41467-022-35156-x (PMC9718836; doi:10.1038/s41467-022-35156-x)

Figure 1E

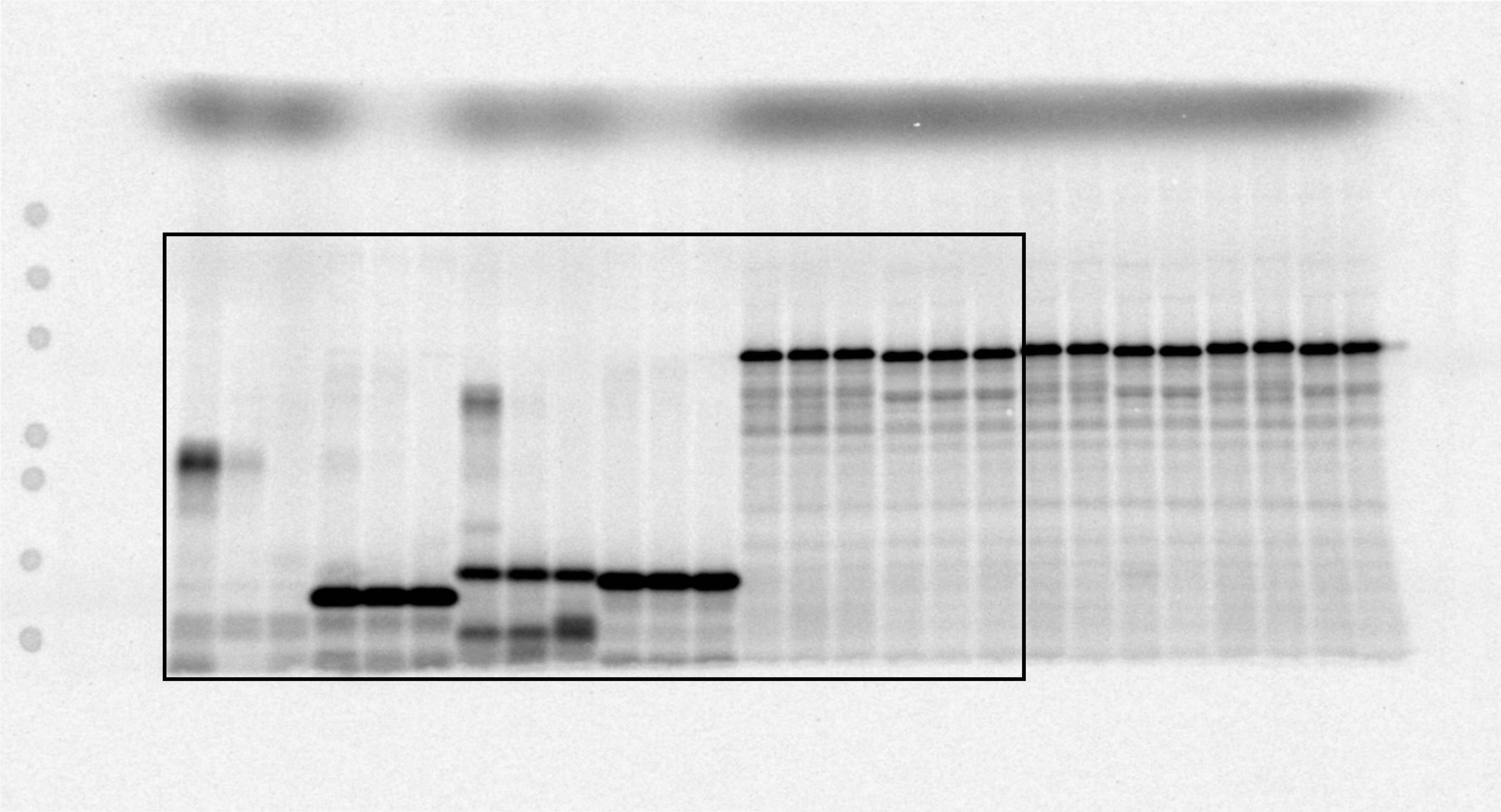

Figure 2B

short exposure

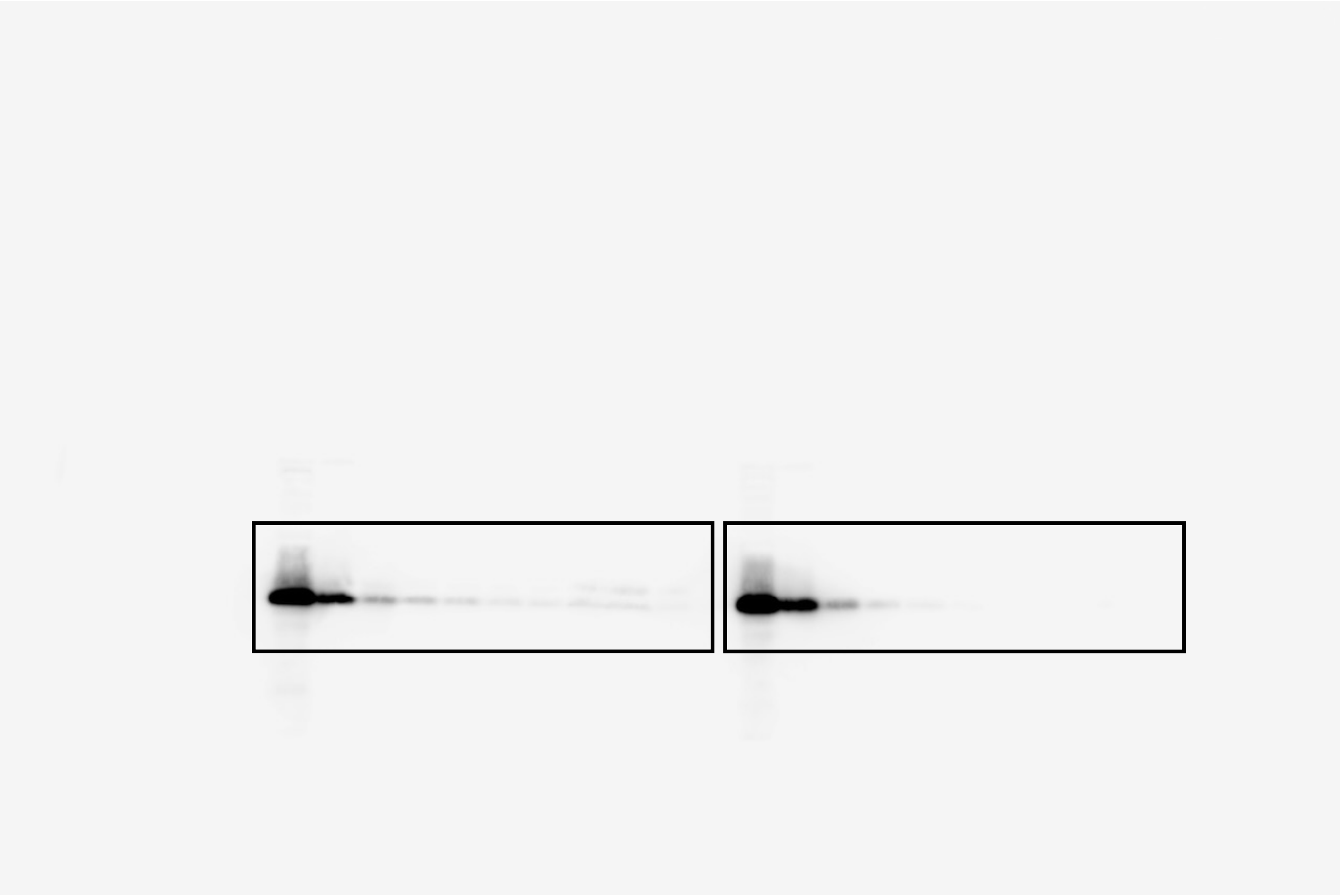

Figure 2B

long exposure

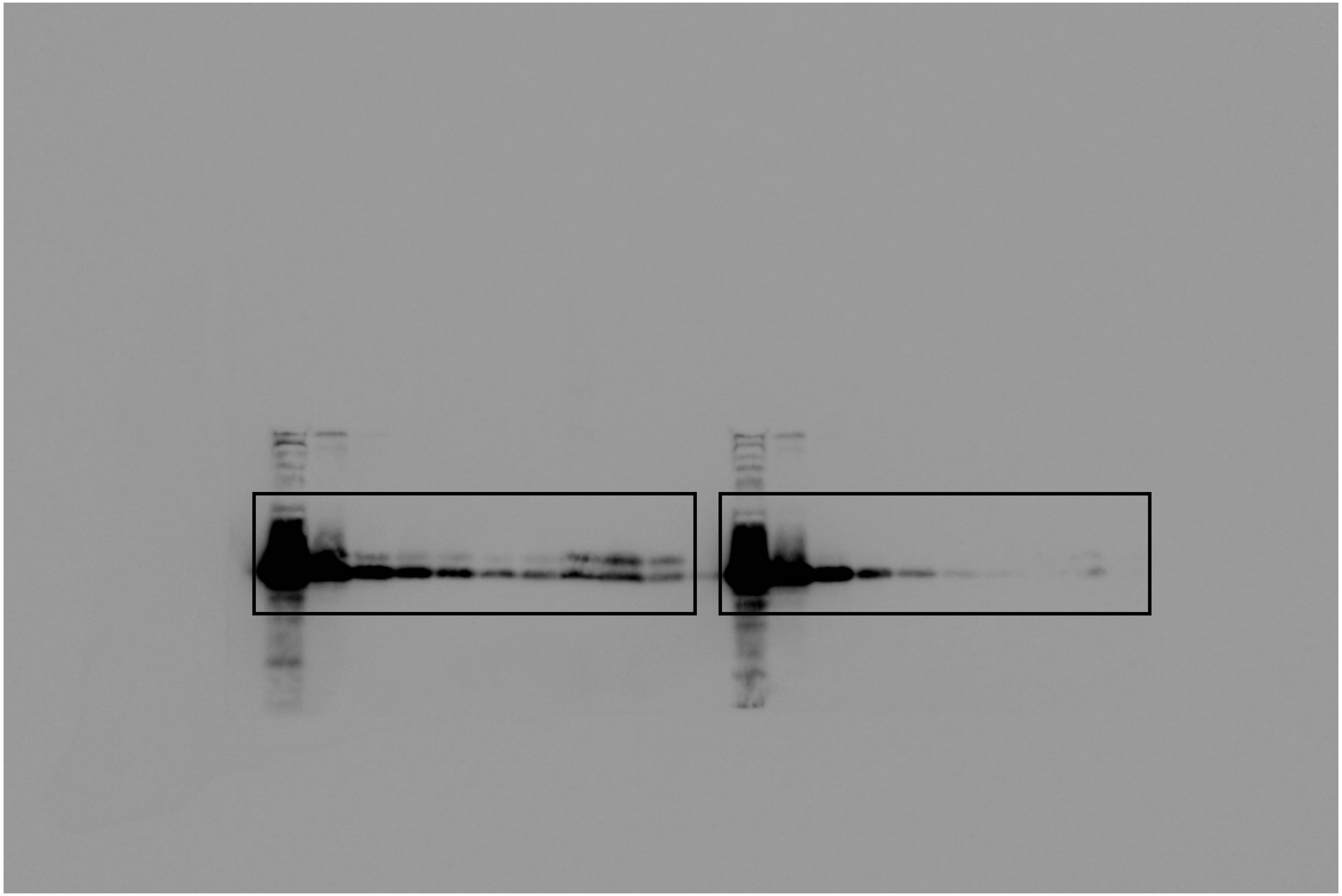

Figure 2D

ATGC marker
lanes 1-8

lanes 9-11

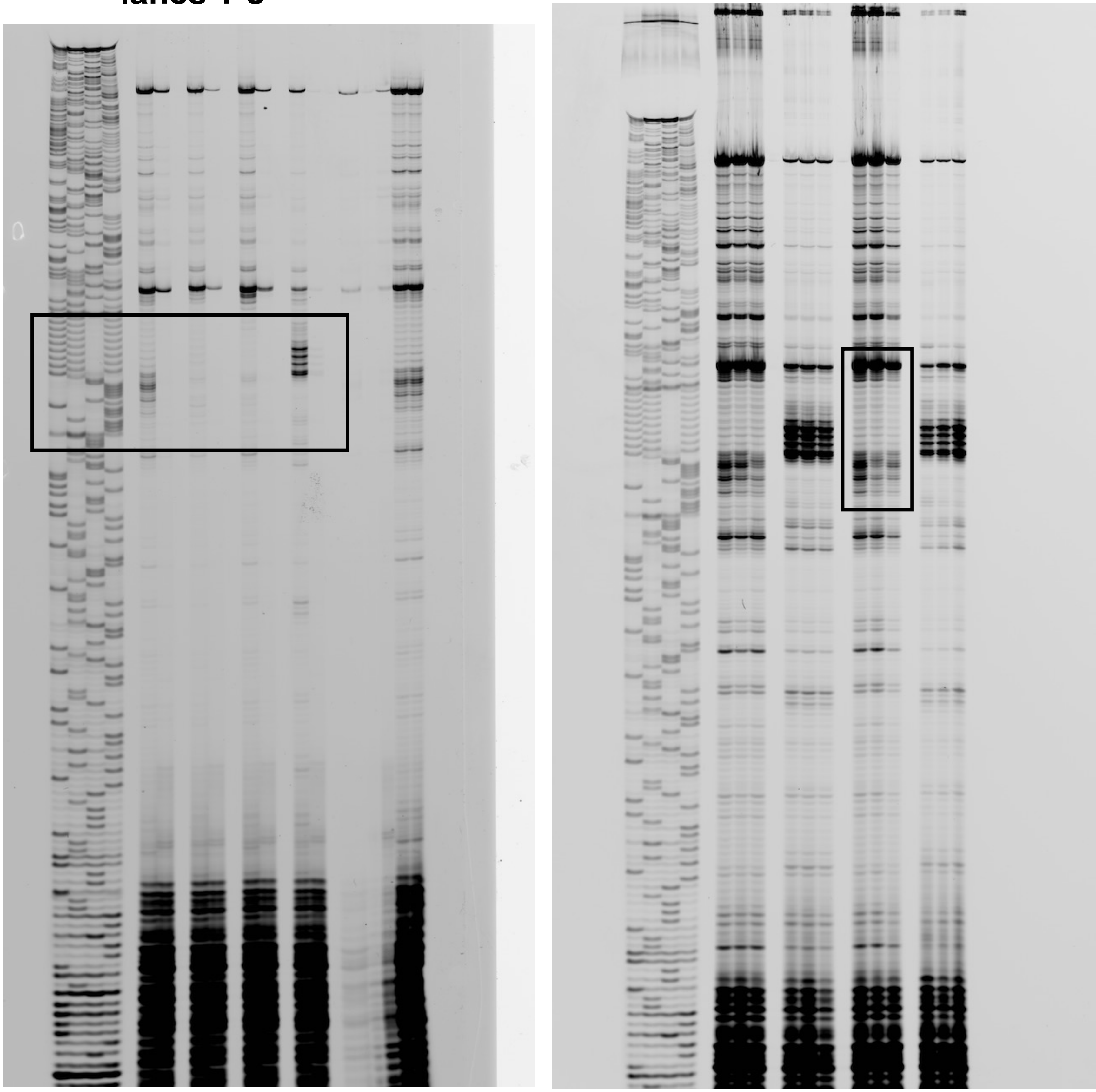

Figure 2E

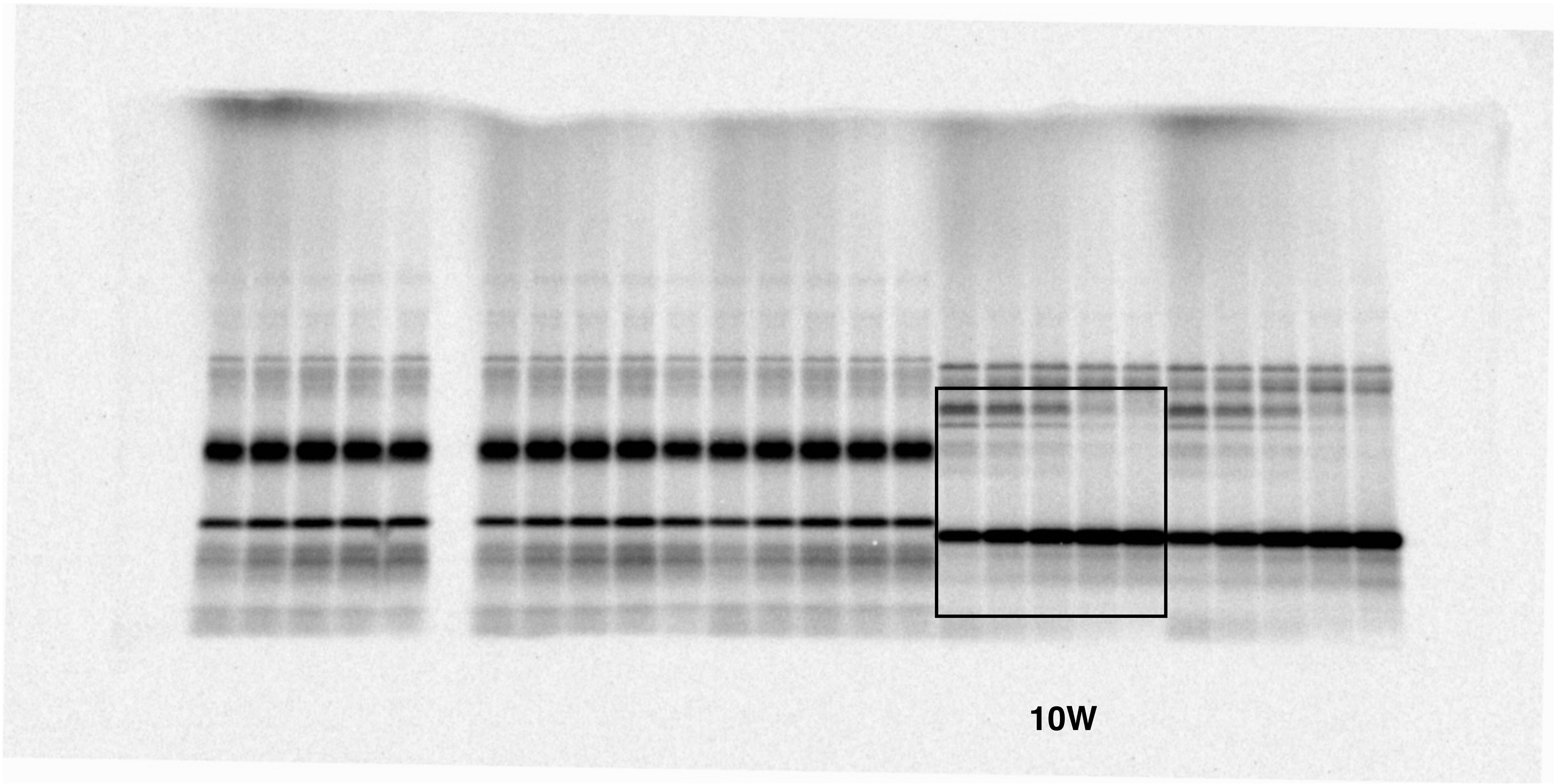

Figure 2E

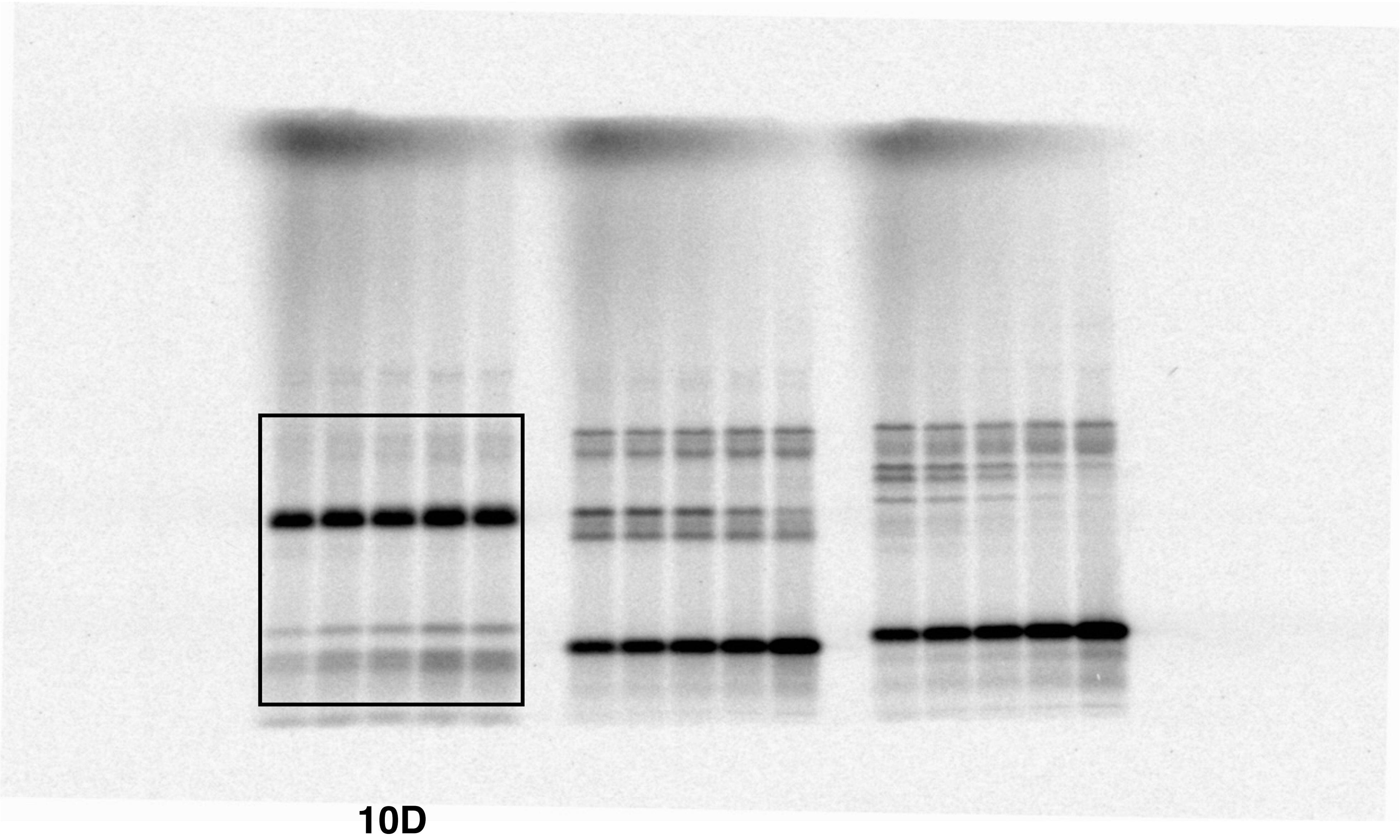

Figure 3D

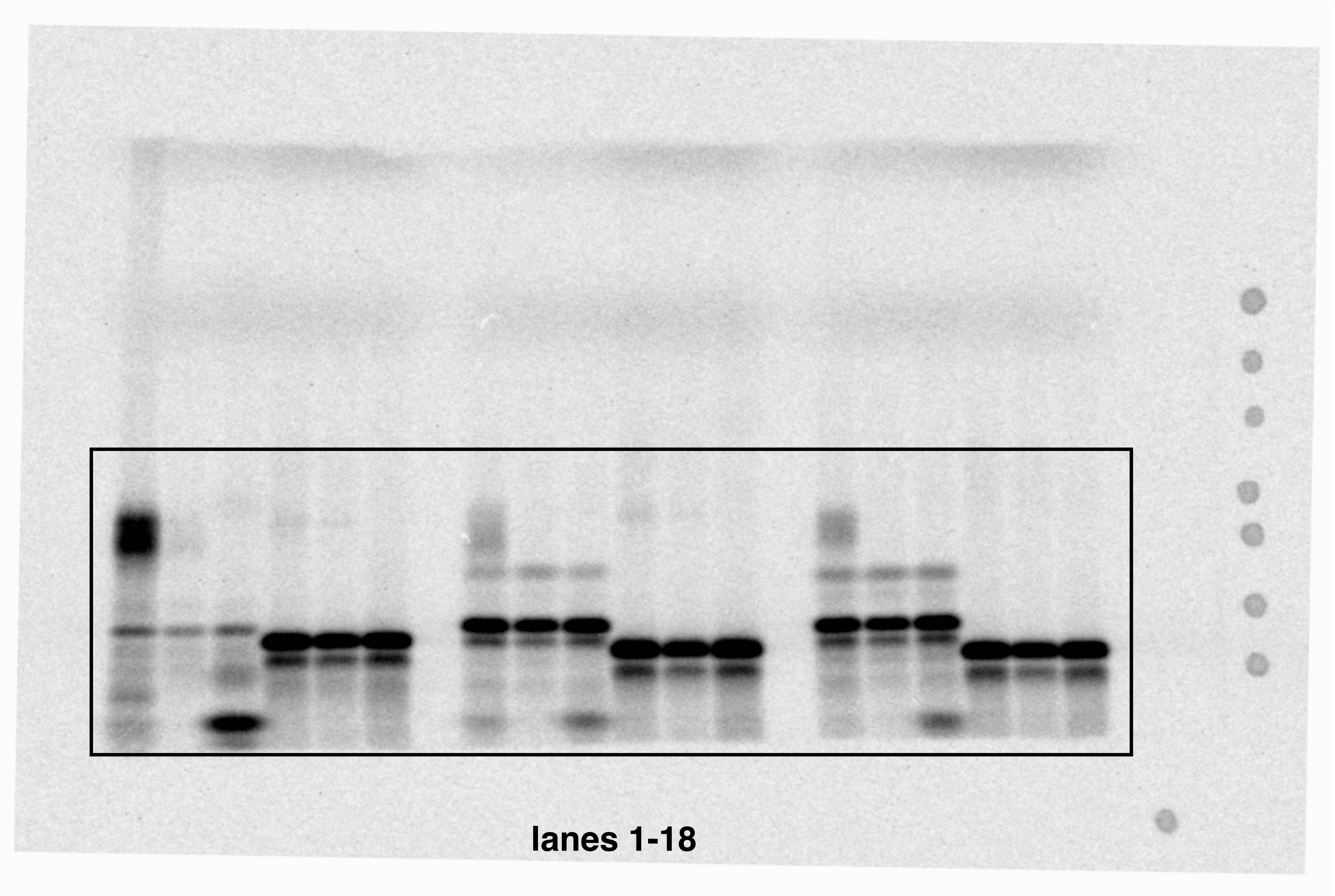

lanes 1-18

Figure 3D

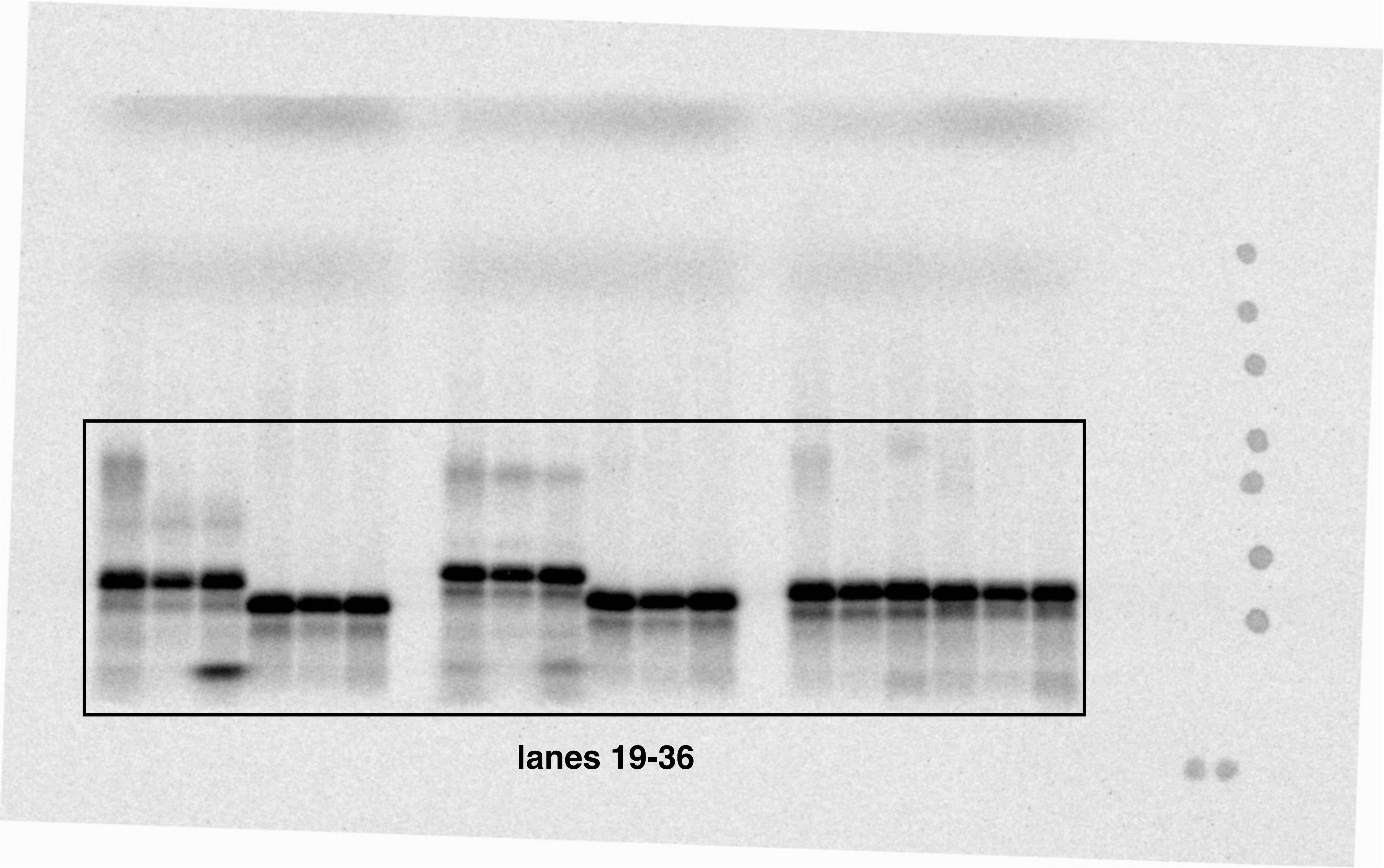

Figure 4C

5S rRNA

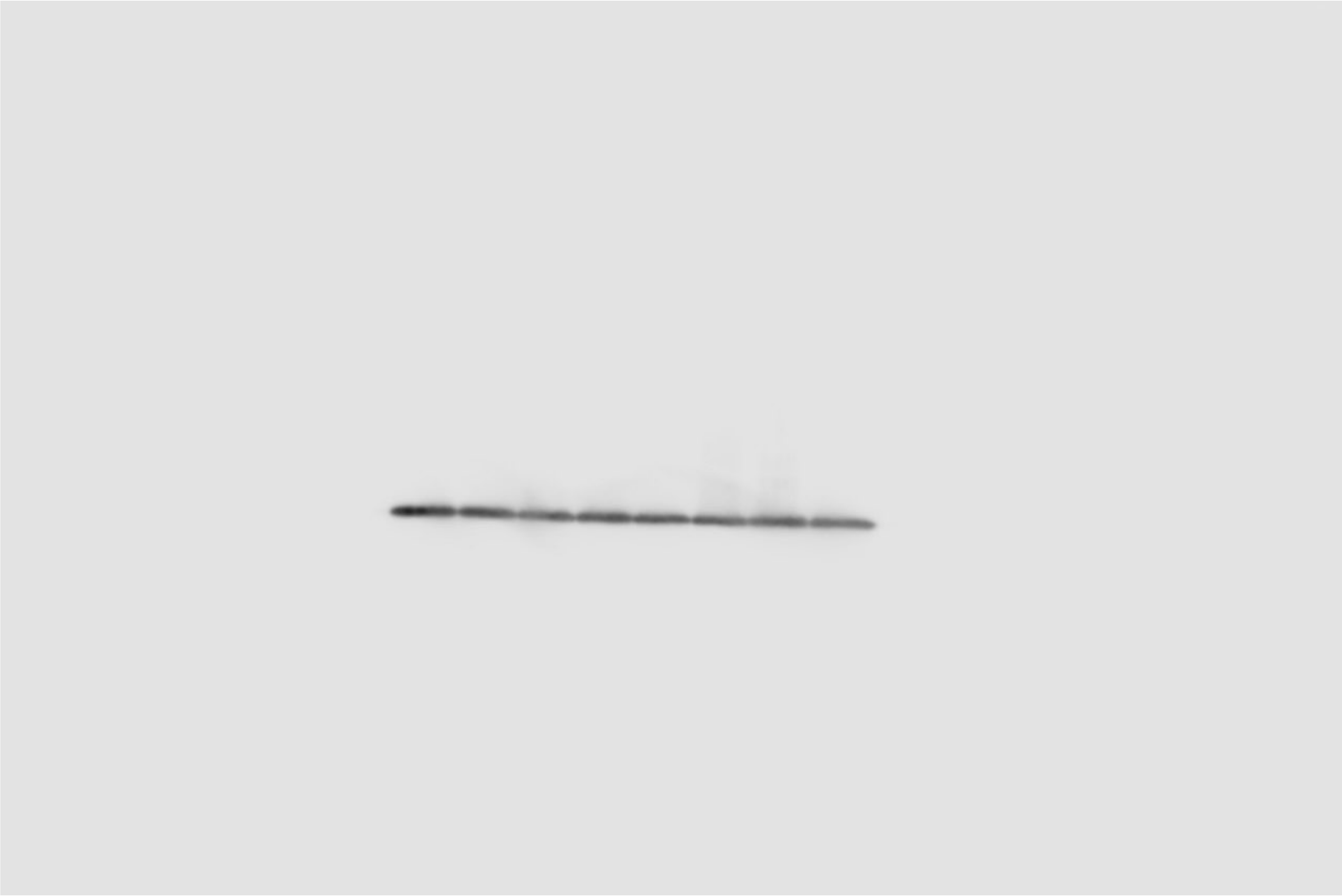

Figure 4C

tRNA_Asn

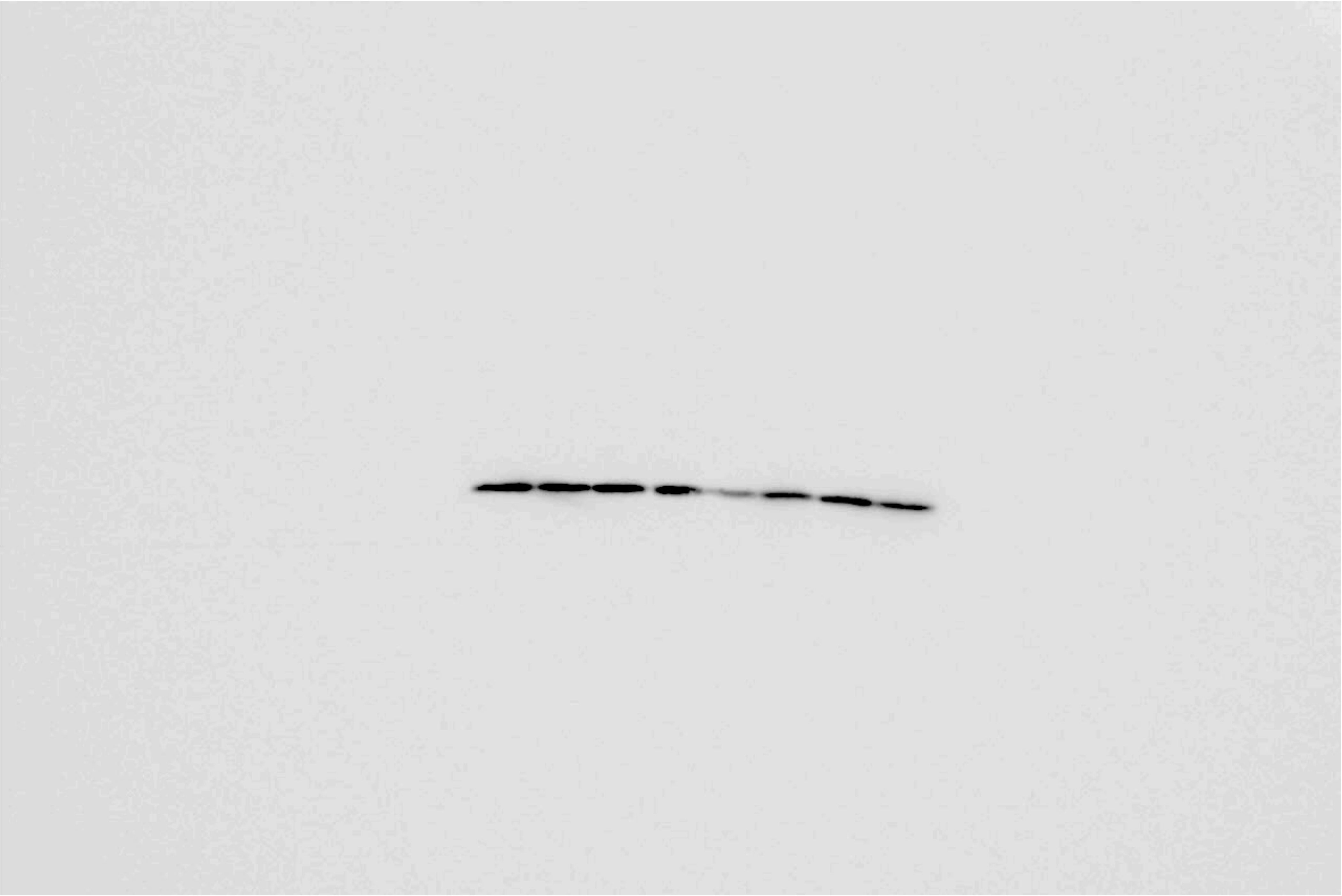

Figure 4C

tRNA_Asp

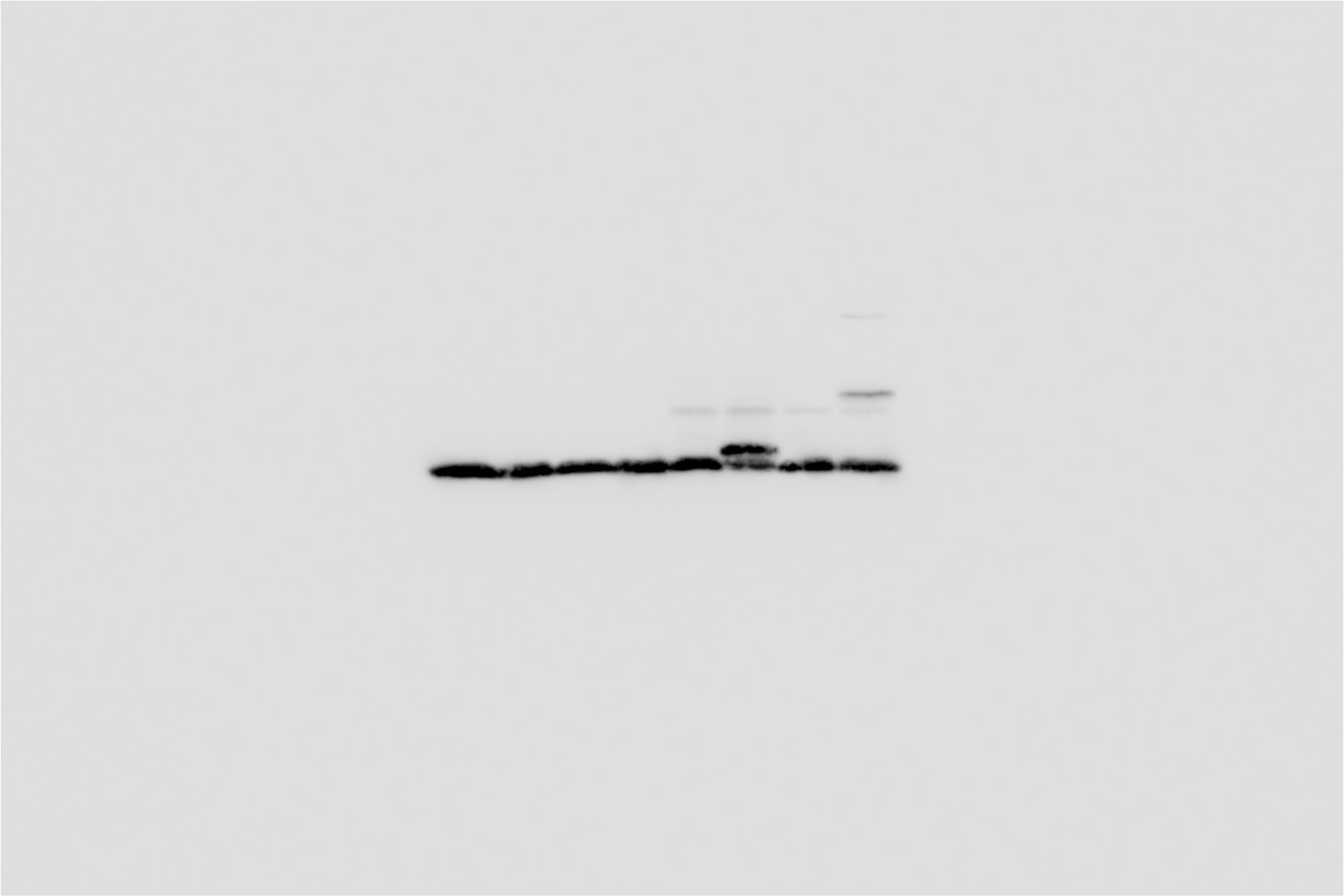

Figure 4D

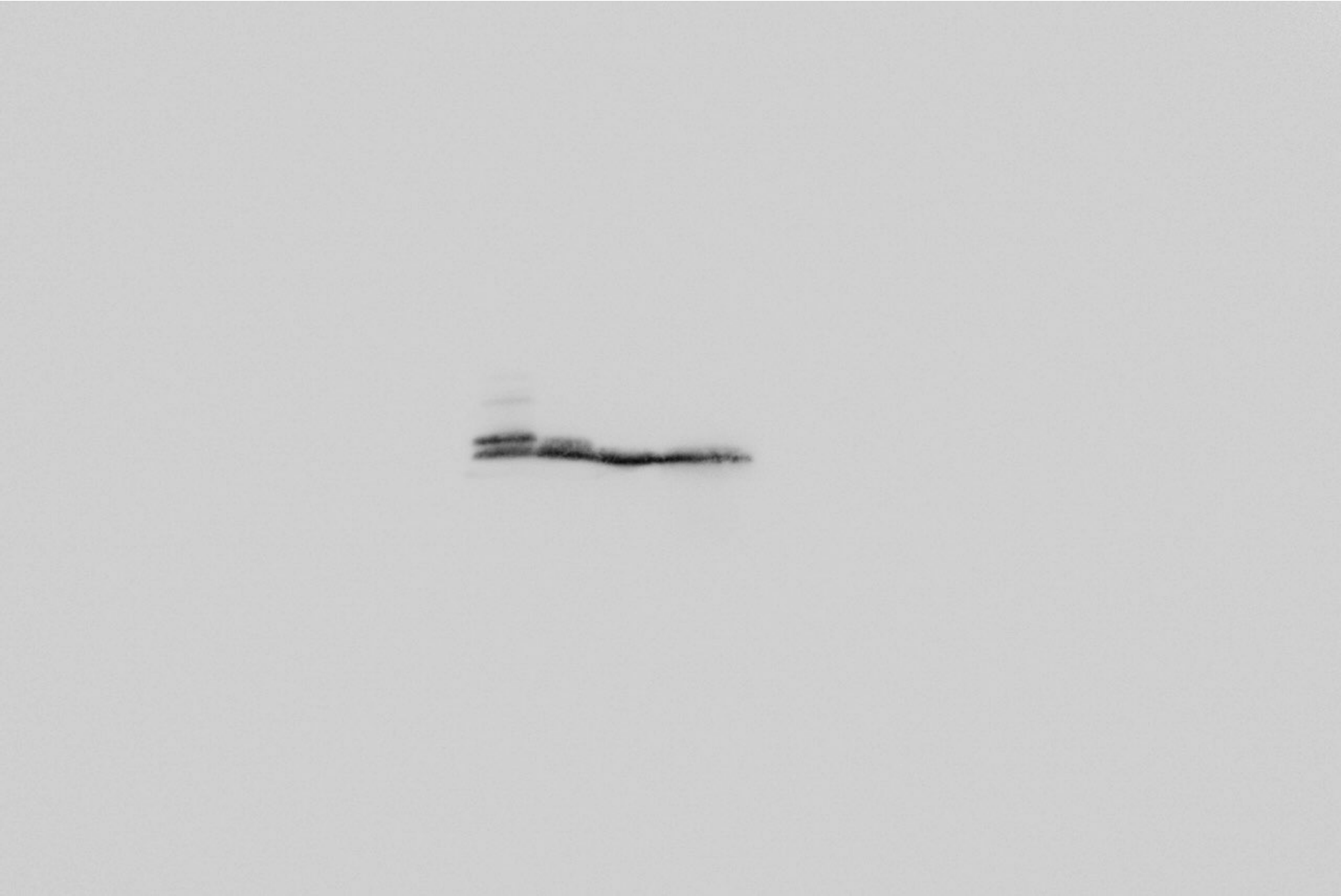

Figure 6B

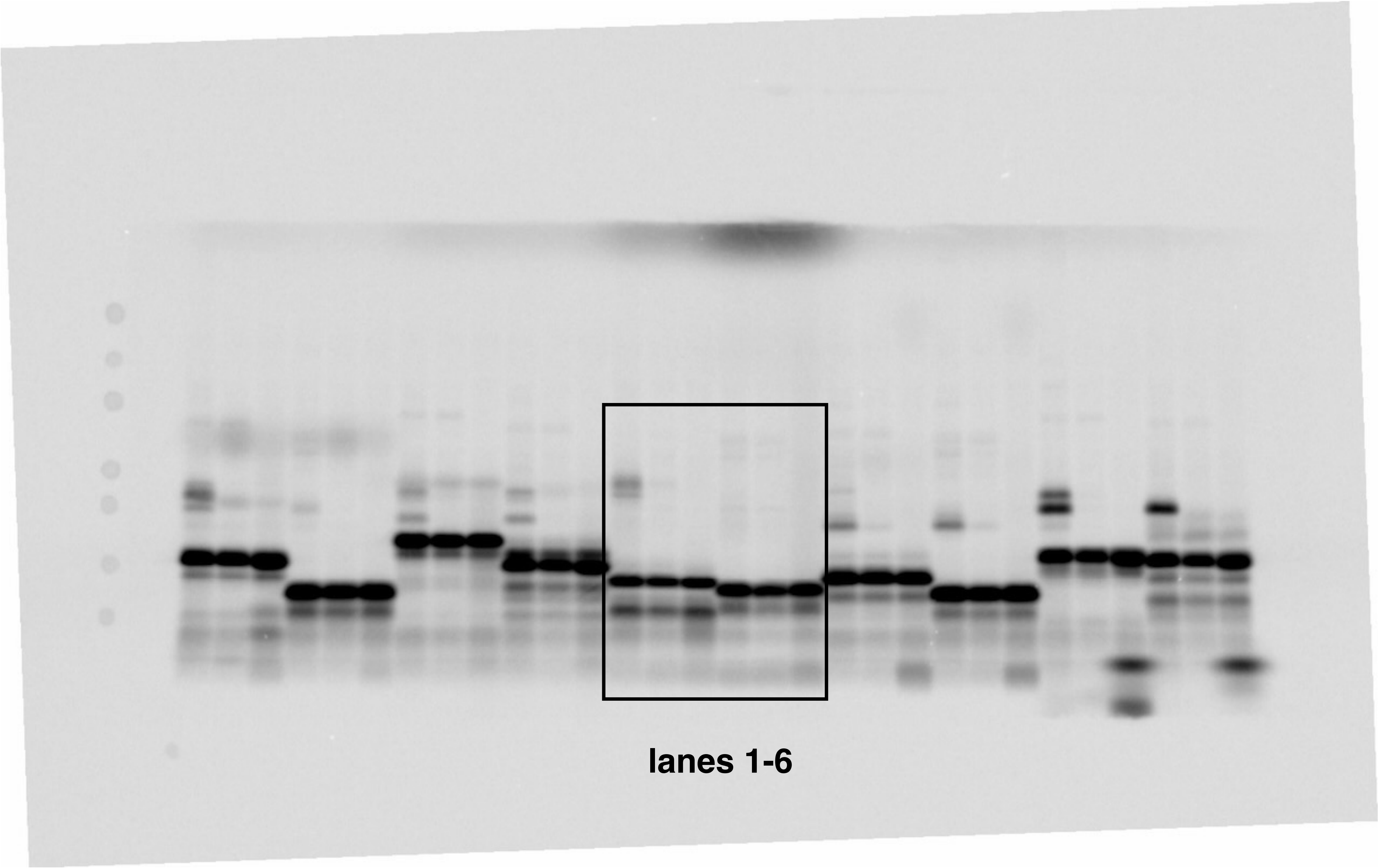

Figure 6B

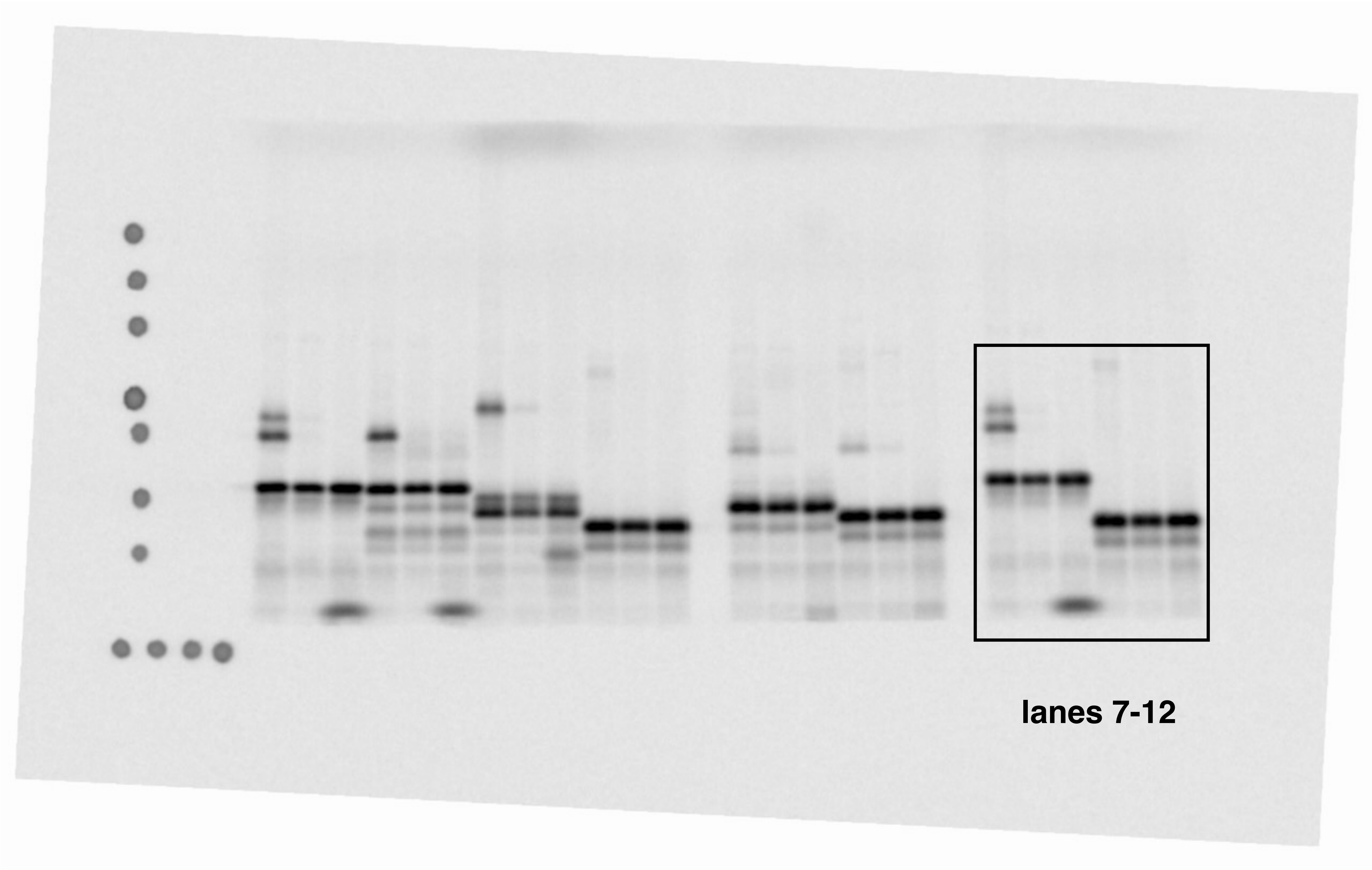

Figure 6B

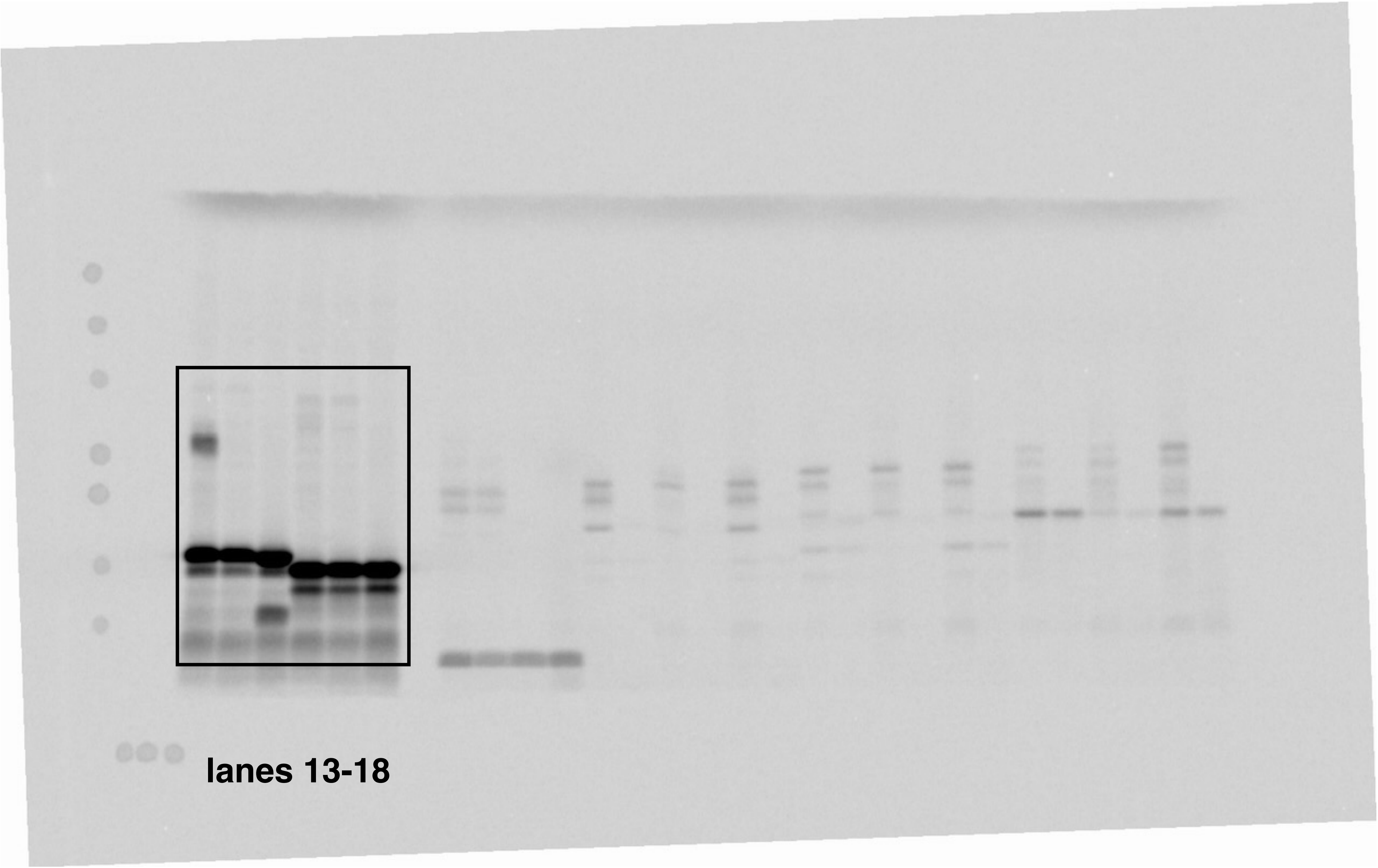

Supplementary Figure S2C

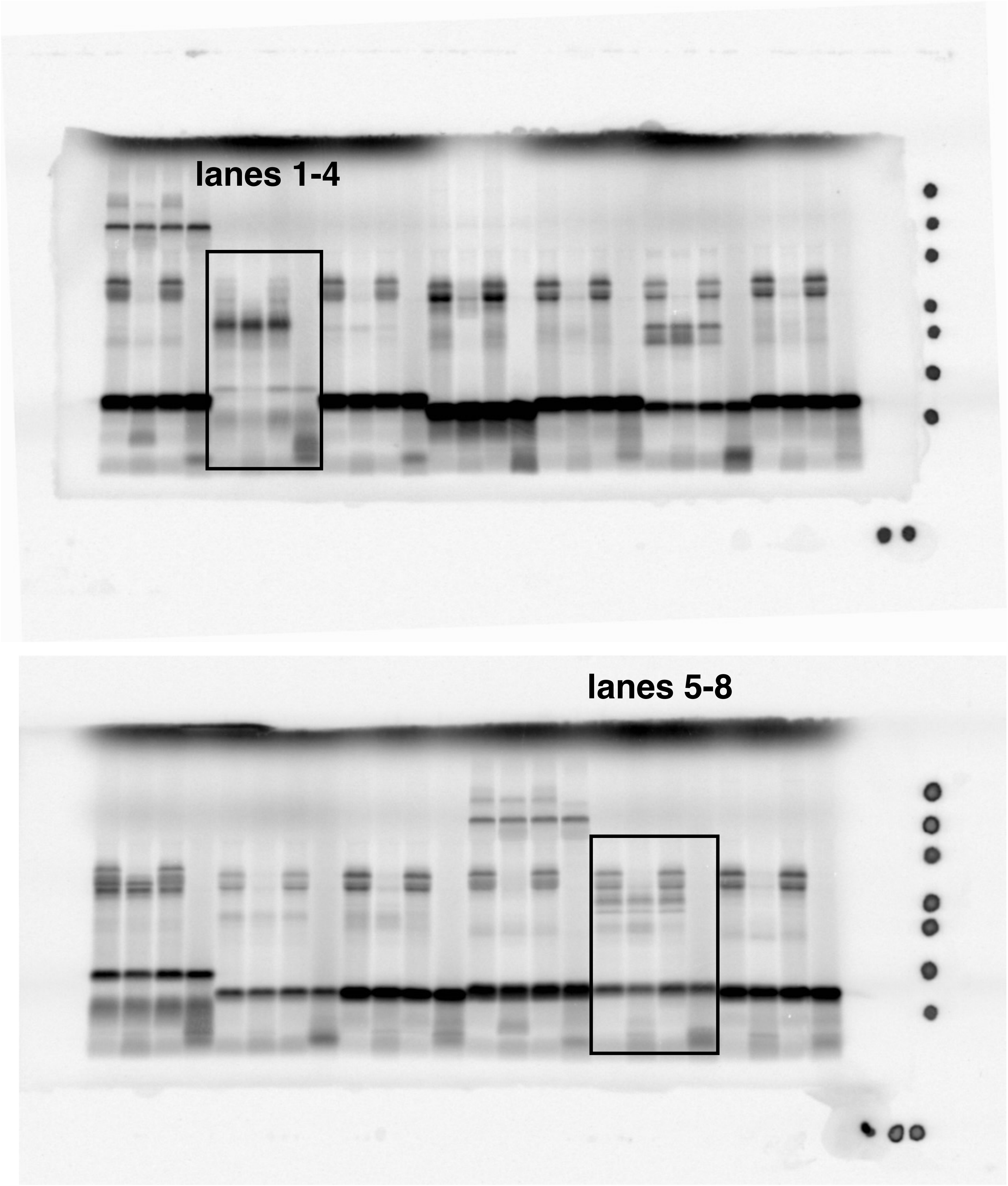

Supplementary Figure S3B

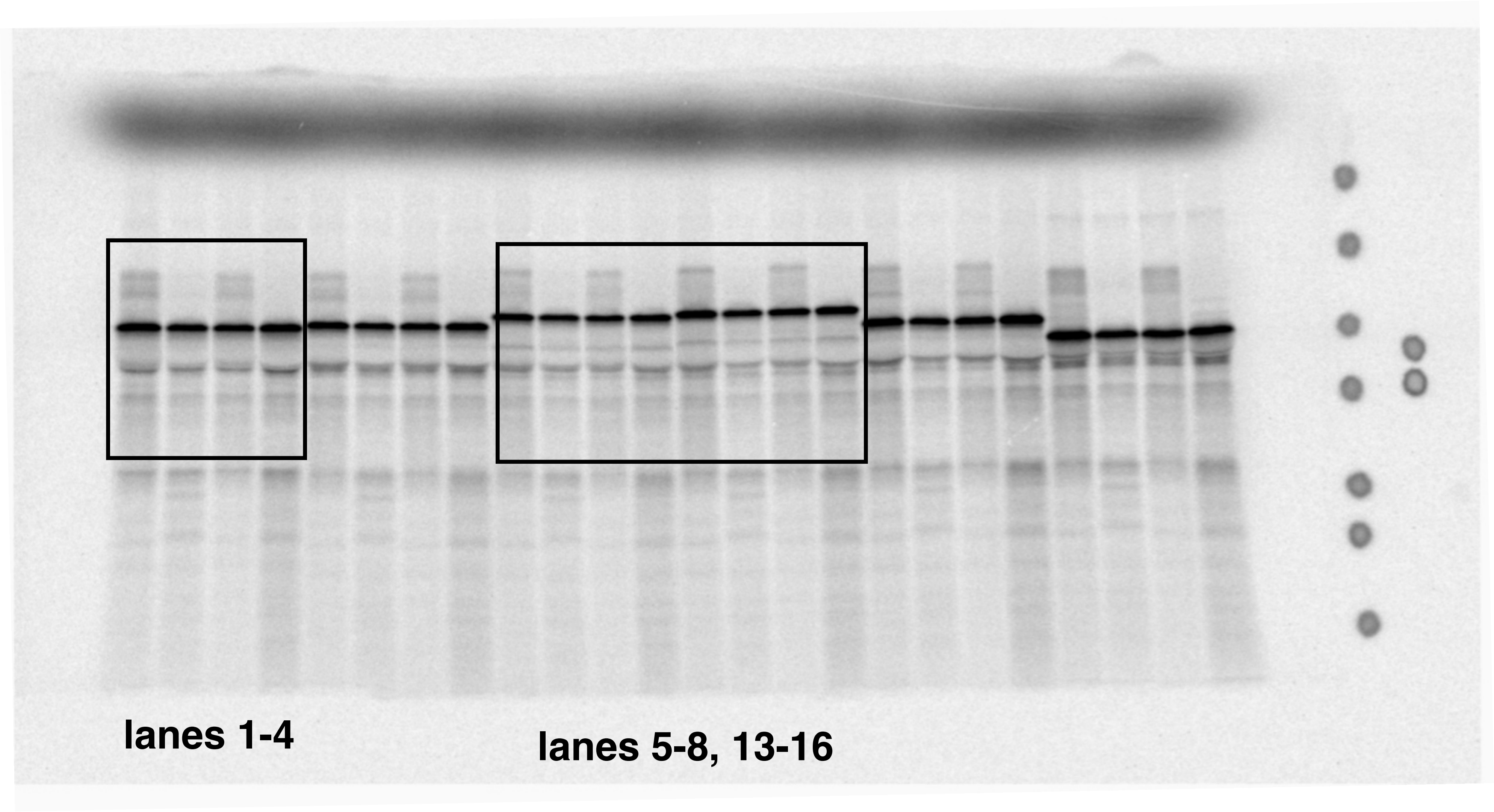

Supplementary Figure S3B

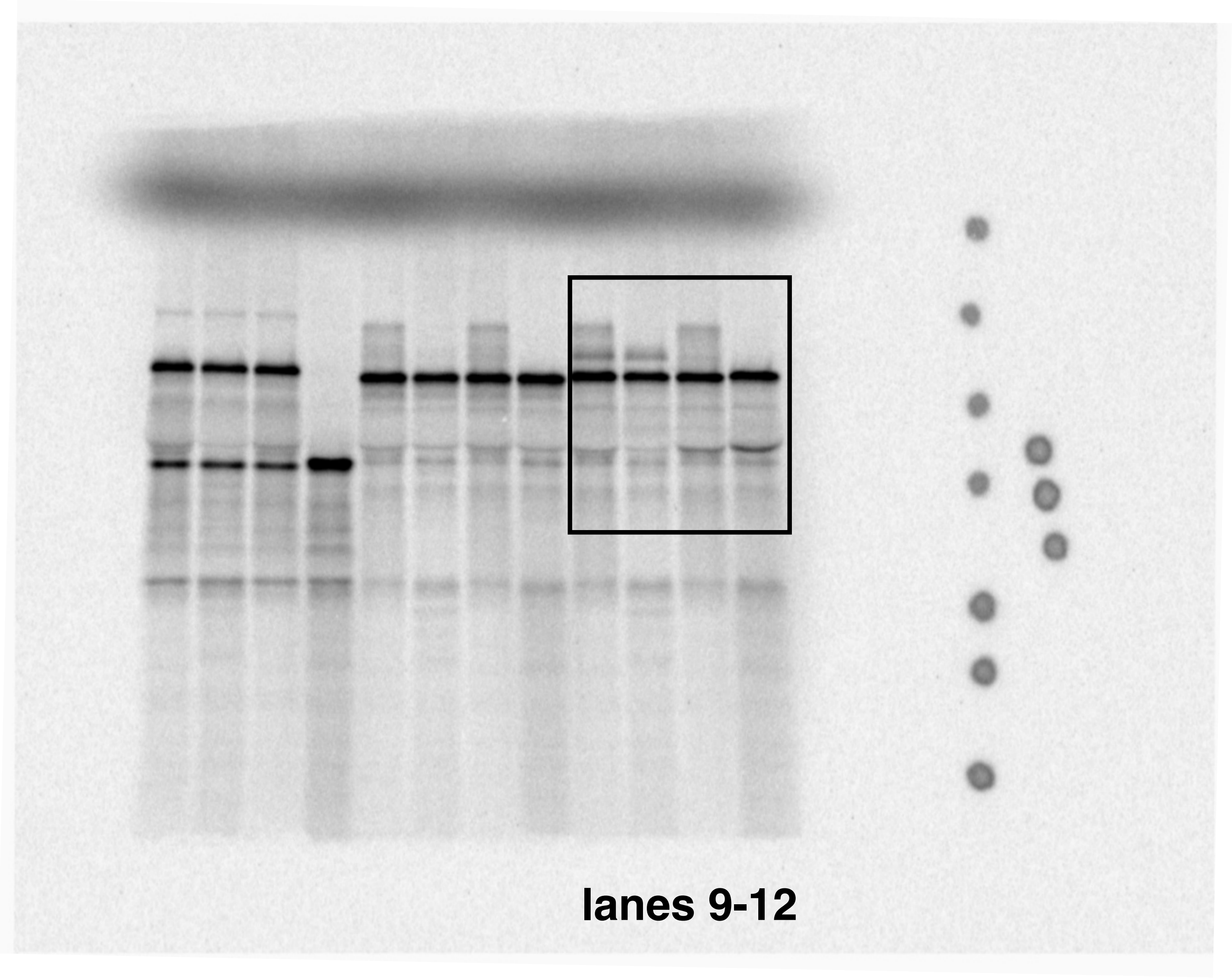

lanes 9-12

Supplementary Figure S3B

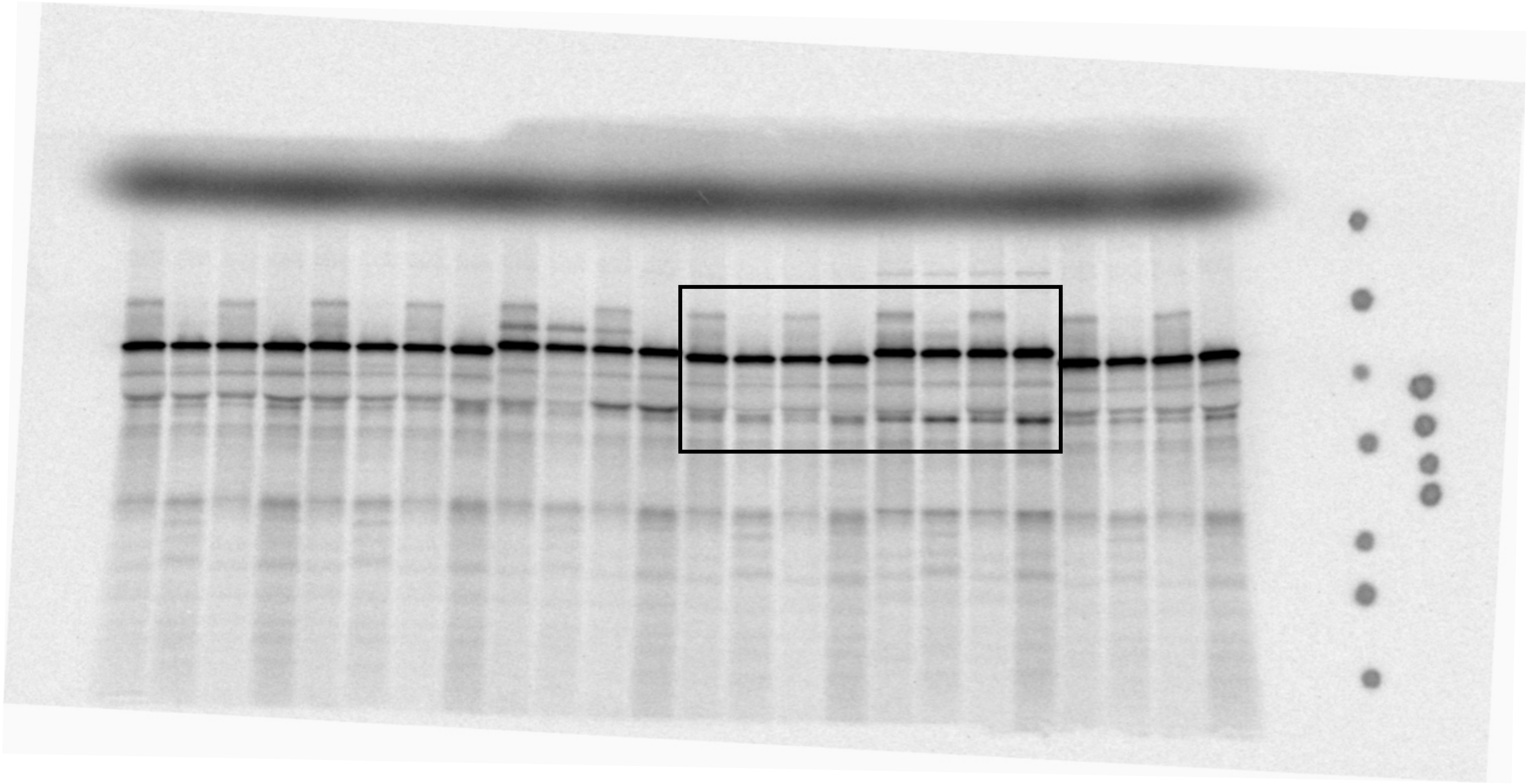

lanes 25-28, 17-20

Supplementary Figure S3B

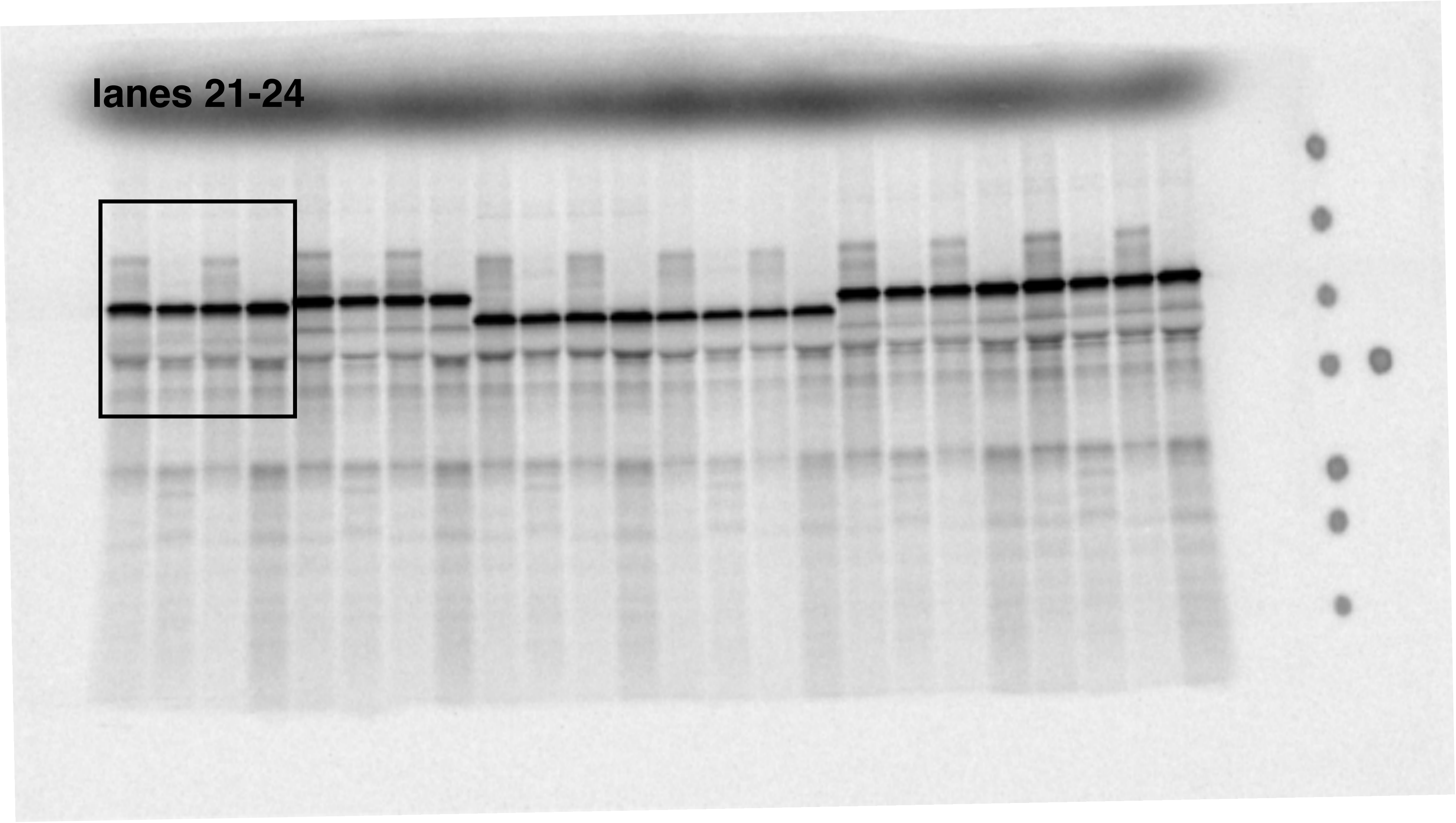

Supplementary Figure S3C

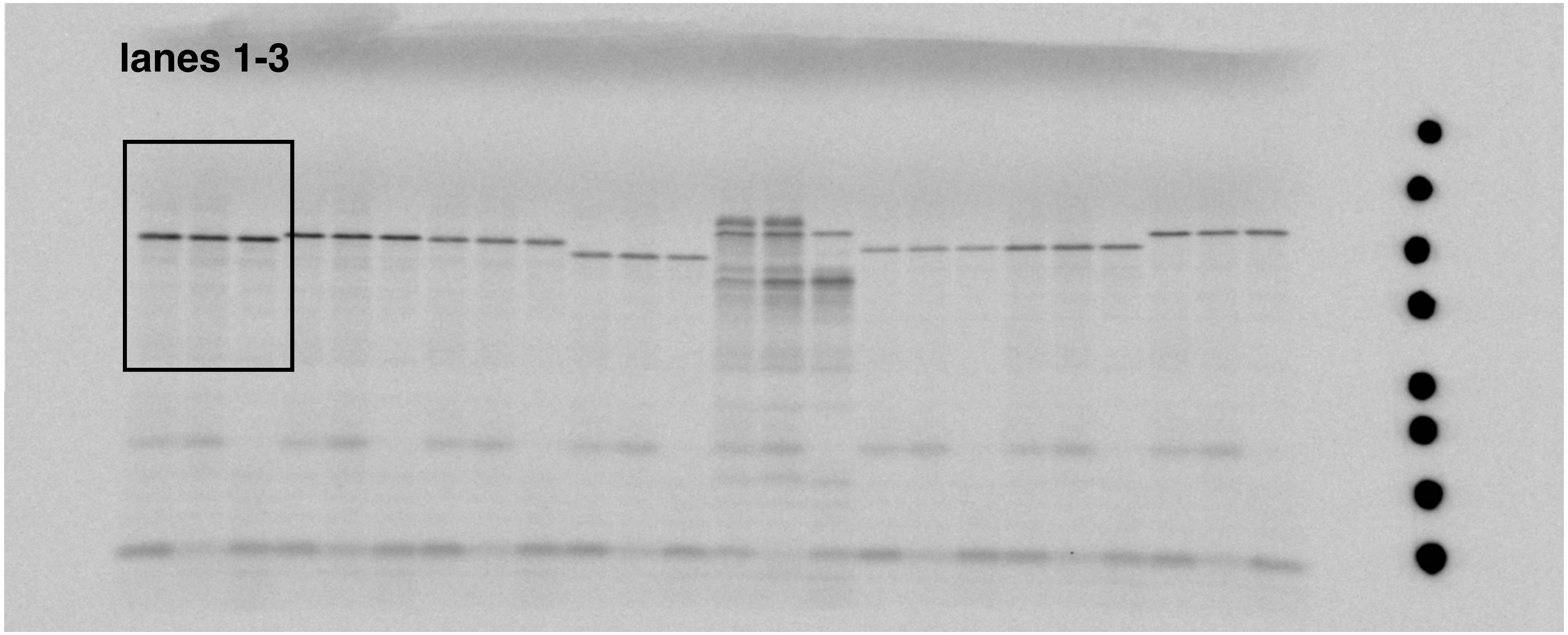

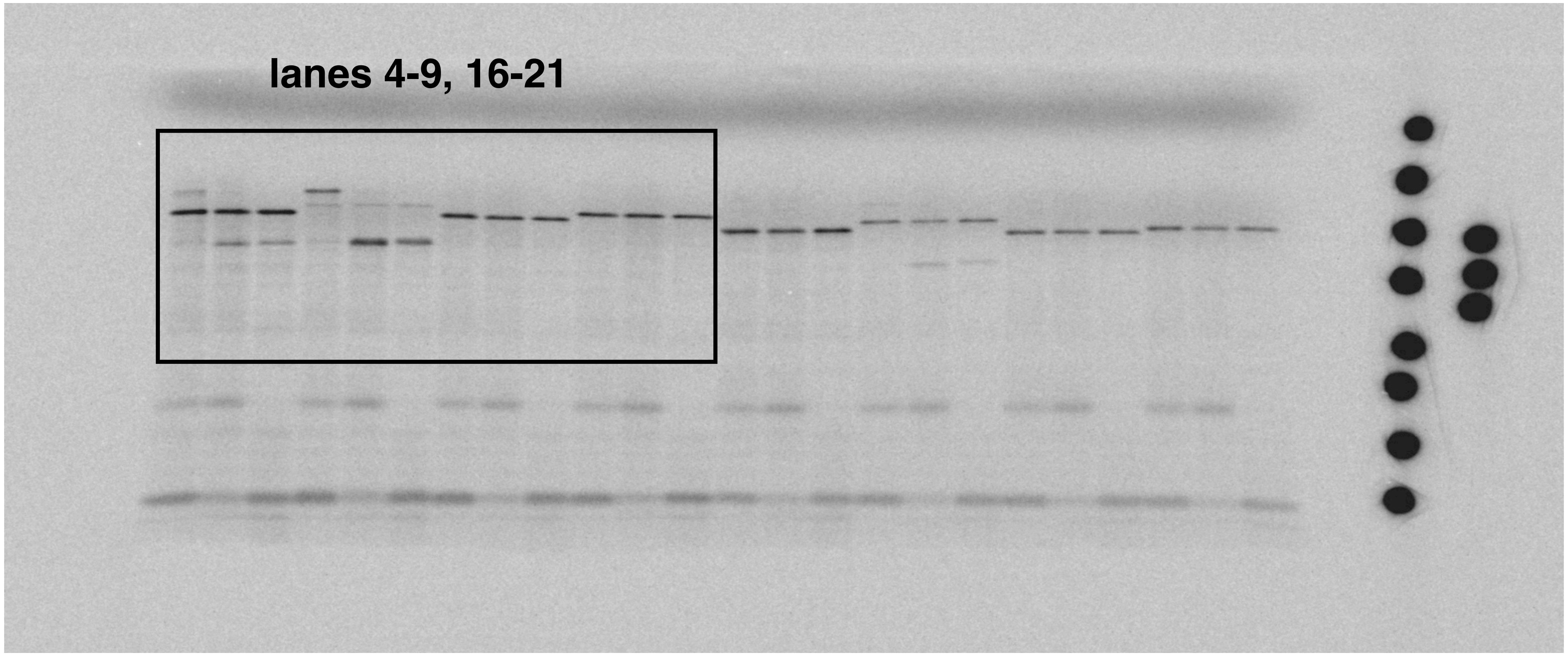

Supplementary Figure S3C

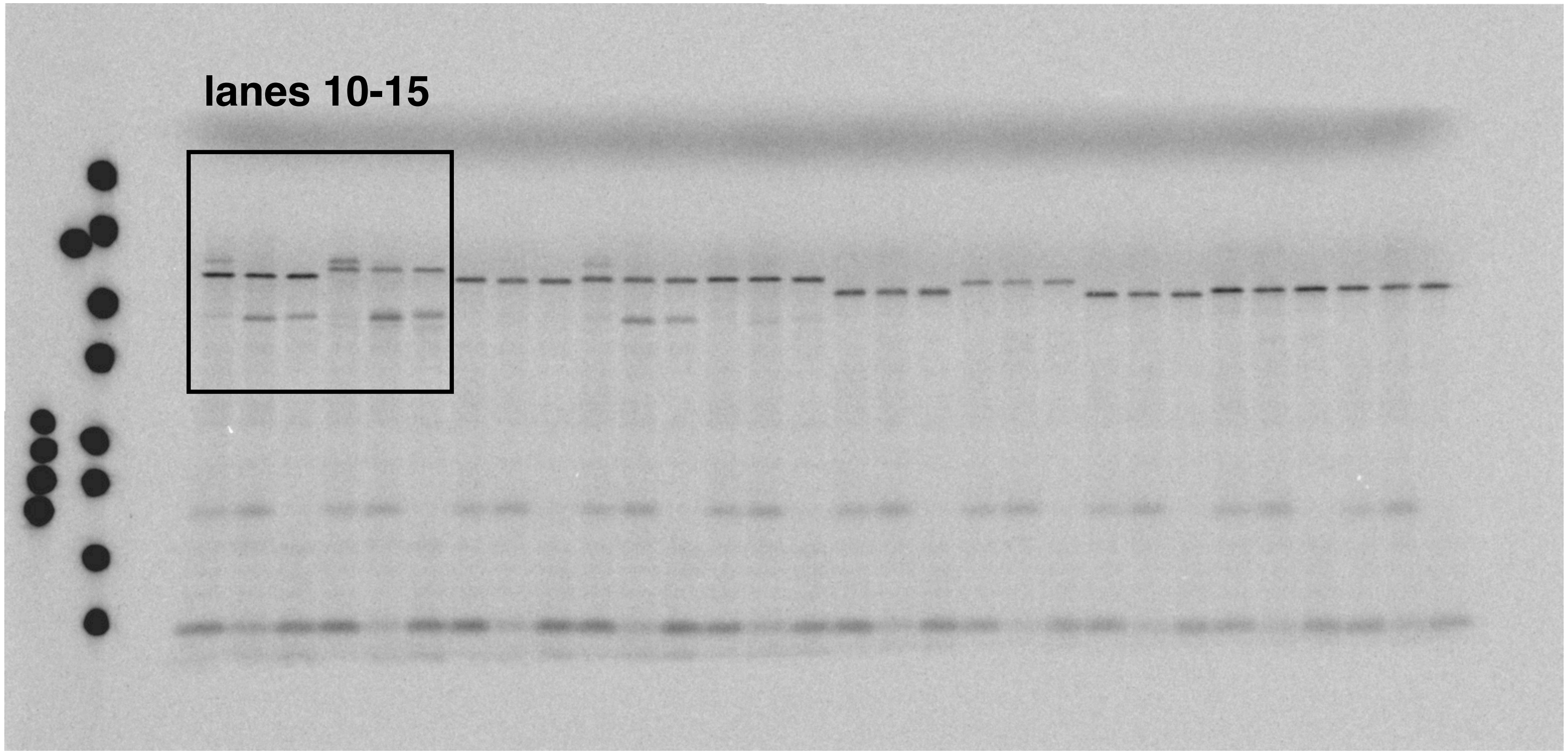

Supplementary Figure S5I

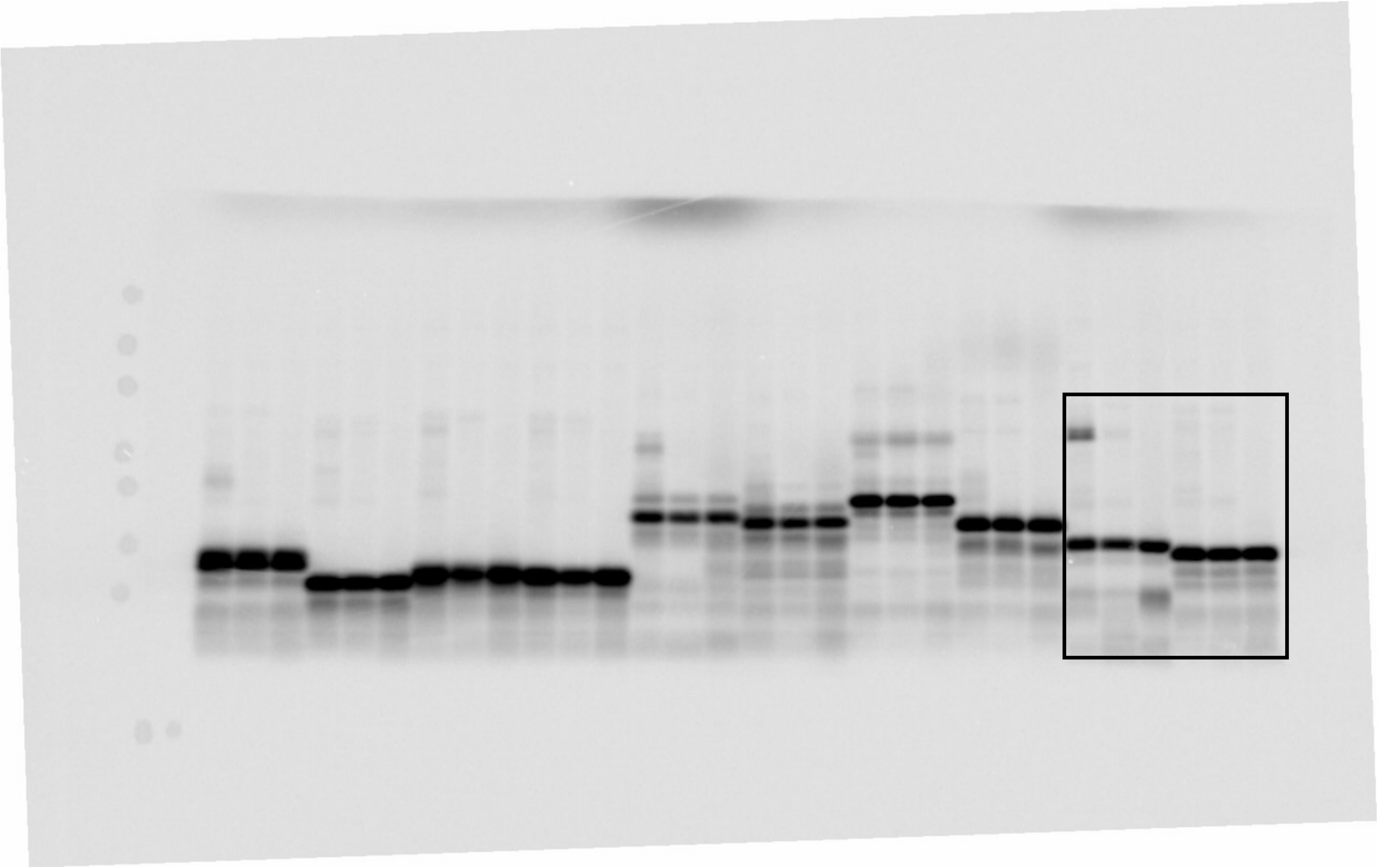

Supplement: Supplementary file 10 — Source Data [file 41467_2022_35156_MOESM10_ESM.zip › Source_data/Gel_raw_image.pdf]
