# Supplementary material for: Nascent peptide-induced translation discontinuation in eukaryotes impacts biased amino acid usage in proteomes
Source: Nat Commun. 2022 Dec 2;13:7451. doi: 10.1038/s41467-022-35156-x (PMC9718836; doi:10.1038/s41467-022-35156-x)
Supplement: Supplementary file 10 — Source Data [file 41467_2022_35156_MOESM10_ESM.zip › Source_data/MS_data_analysis.zip!/MS_data_analysis/YIto_paper_ratio_less_40aa_from_Nterm_late_log_rem_common.pdf]

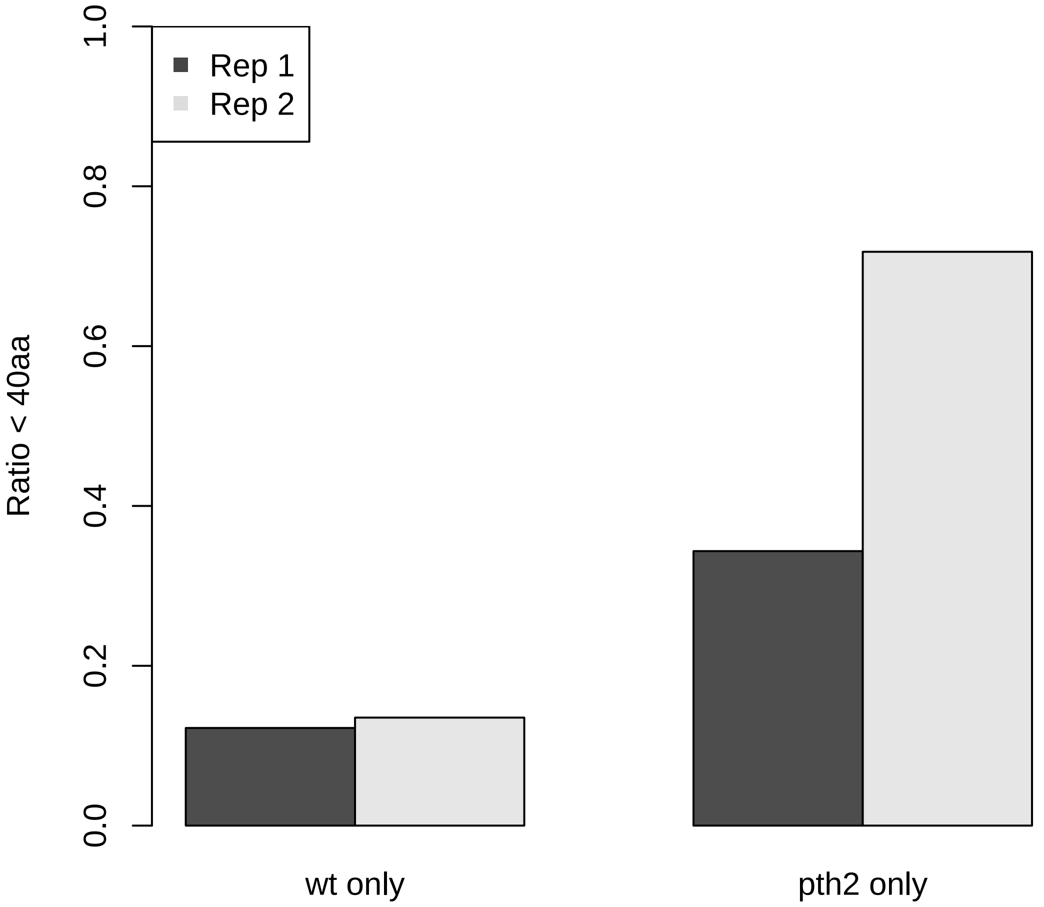

Ratio < 40aa
1.0
0.8
0.6
0.4
0.2
0.0
Rep 1
Rep 2
wt only
pth2 only
